# Associations between gut microbiota and Alzheimer’s disease, major depressive disorder, and schizophrenia

**DOI:** 10.1186/s12974-020-01961-8

**Published:** 2020-10-02

**Authors:** Zhenhuang Zhuang, Ruotong Yang, Wenxiu Wang, Lu Qi, Tao Huang

**Affiliations:** 1 Department of Epidemiology & Biostatistics, School of Public Health, Peking University, 38 Xueyuan Road, Beijing, 100191 China; 2 Department of Epidemiology, School of Public Health and Tropical Medicine, Tulane University, New Orleans, LA USA; 3 Department of Nutrition, Harvard T.H. Chan School of Public Health, Boston, MA USA; 4 Department of Global Health, School of Public Health, Peking University, Beijing, 100191 China; 5 Key Laboratory of Molecular Cardiovascular Sciences (Peking University), Ministry of Education, Beijing, 100191 China; 6 Center for Intelligent Public Health, Institute for Artificial Intelligence, Peking University, Beijing, 100191 China

**Keywords:** Gut microbiota, Neuropsychiatric disorder, Mendelian randomization, Genetic association, Causality

## Abstract

**Background:**

Growing evidence has shown that alterations in the gut microbiota composition were associated with a variety of neuropsychiatric conditions. However, whether such associations reflect causality remains unknown. We aimed to reveal the causal relationships among gut microbiota, metabolites, and neuropsychiatric disorders including Alzheimer’s disease (AD), major depressive disorder (MDD), and schizophrenia (SCZ).

**Methods:**

A two-sample bi-directional Mendelian randomization analysis was performed by using genetic variants from genome-wide association studies as instrumental variables for gut microbiota, metabolites, AD, MDD, and SCZ, respectively.

**Results:**

We found suggestive associations of host-genetic-driven increase in *Blautia* (OR, 0.88; 95%CI, 0.79–0.99; *P* = 0.028) and elevated γ-aminobutyric acid (GABA) (0.96; 0.92–1.00; *P* = 0.034), a downstream product of *Blautia*-dependent arginine metabolism, with a lower risk of AD. Genetically increased *Enterobacteriaceae family* and *Enterobacteriales order* were potentially associated with a higher risk of SCZ (1.09; 1.00–1.18; *P* = 0.048), while *Gammaproteobacteria class* (0.90; 0.83–0.98; *P* = 0.011) was related to a lower risk for SCZ. Gut production of serotonin was potentially associated with an increased risk of SCZ (1.07; 1.00–1.15; *P* = 0.047). Furthermore, genetically increased *Bacilli class* was related to a higher risk of MDD (1.07; 1.02–1.12; *P* = 0.010). In the other direction, neuropsychiatric disorders altered gut microbiota composition.

**Conclusions:**

These data for the first time provide evidence of potential causal links between gut microbiome and AD, MDD, and SCZ. GABA and serotonin may play an important role in gut microbiota-host crosstalk in AD and SCZ, respectively. Further investigations in understanding the underlying mechanisms of associations between gut microbiota and AD, MDD, and SCZ are required.

## Background

The human intestine comprises a very complex group of gut microbiota, which influence the risk of neuropsychiatric disorders [1, 2]. Accumulating evidence has suggested that microbiota metabolites such as neurotransmitters and short-chain fatty acids (SCFAs) may play a central role in microbiota-host crosstalk that regulates the brain function and behavior [3, 4]. Therefore, to understand the mechanism of the gut-brain axis in neuropsychiatric disorders may have clinical benefits.

Observational studies, most of case-control designs, have shown differences in the composition of the gut microbiota between healthy individuals and patients with neuropsychiatric disorders such as Alzheimer’s disease (AD), major depression disorder (MDD), and schizophrenia (SCZ); however, such associations substantially differed across studies [5–7]. Noteworthy, genome-based metabolic modeling of the human gut microbiota revealed that several genera have predictive capability to produce or consume neurotransmitters (called microbial neurotransmitters) such as γ-aminobutyric acid (GABA) and serotonin [8, 9], which have been consistently shown to played a key role in the regulation of brain function [10, 11]. A meta-analysis of 35 observational studies reported that increased GABA levels were associated with a lower risk of AD [12]. In addition, a previous study (*n* = 40) reported that plasma serotonin was lower and platelet serotonin was higher in SCZ patients compared with controls [13], while another study showed that lower platelet serotonin concentrations were associated with depressive symptoms of SCZ (*n* = 364) [14]. There is no doubt that these small observational studies were susceptible to confounding bias and reverse causation. It is crucial to elucidate whether such associations reflect causal relations or spurious correlations due to bias.

Mendelian randomization (MR), which overcomes the bias due to confounding and reverse causation abovementioned, has been widely used to assess causal relationships by exploiting genetic variants as instrumental variables of the exposure [15]. Recent genetic studies have demonstrated that the host genetic variants influence the gut microbiota composition [16–18]. Thus, such findings allowed us to deploy an MR approach to infer the mutually causal relations of gut microbiota and metabolites with neuropsychiatric disorders.

Therefore, we for the first time applied a two-sample bi-directional MR approach to detect causal relationships among gut microbiota, metabolites, and diverse forms of neuropsychiatric disorders including AD, SCZ, and MDD.

## Methods

### Study design overview

We employed a two-sample bi-directional MR approach to investigate the causal relationships among gut microbiota, metabolites, and AD, MDD, or SCZ using summary-level data from large genome-wide association studies (GWASs) for gut microbiota and AD, MDD, or SCZ. Ethical approval for each study included in the MR analysis can be found in the original articles [19–23].

### Data sources and instruments

#### Gut microbiota

We leveraged summary statistics from a GWAS of gut microbiota conducted among two independent but geographically matched cohorts of European ancestry (*n* = 1812) using 16S rRNA gene sequencing (Table 1) [19], which yielded a total of 38 and 374 identified phyla and genera respectively. The GWAS defined a “core measurable microbiota” after removing rare bacteria and investigating associations between host genetic variants and specific bacterial traits, including 40 operational taxonomic units (OTUs) and 58 taxa ranging from the genus to the phylum level. Accordingly, the GWAS further identified 54 genome-wide significant associations involving 40 loci and 22 bacterial traits (meta-analysis *P* < 5 × 10^−8^). We selected single nucleotide polymorphisms (SNPs) at thresholds for genome-wide significance (*P* < 5 × 10^−8^) from this GWASs as genetic instruments (Table S1).

**Table 1:** Description of gut microbiota, metabolites, and neuropsychiatric disorders

Traits	Consortium or study	Sample size	Populations	Journal	Year
**Gut**					
Gut microbiota	PopGen/FoCus	1812 individuals	European	Nat Genet.	2016
Gut metabolites	FHS	2076 individuals	European	Cell Metab.	2013
**Neuropsychiatric disorders**					
Alzheimer’s disease	IGAP^a^	25,580 cases and 48,466 controls	European	Nat Genet.	2013
Major depression disorder	PGC29/deCODE/GenScotland/GERA/iPSYCH/UK Biobank/23andMeD	135,458 cases and 344,901 controls	European	Nat Genet.	2018
Schizophrenia	Sweden/PGC	21,246 cases and 38,072 controls	European	Nat Genet.	2013

*FoCus* Food-Chain Plus, *GERA* Genetic Epidemiology Research on Adult Health and Aging, *PGC* Psychiatric Genomics Consortium

^a^ IGAP includes the Alzheimer’s Disease Genetics Consortium (ADGC), the Cohorts for Heart and Aging Research in Genomic Epidemiology consortium (CHARGE), the European Alzheimer’s disease Initiative (EADI), and the Genetic and Environmental Risk in Alzheimer’s disease consortium (GERAD)

#### Gut microbial metabolites

Considering the important roles of gut microbiota-derived metabolites in microbiota-host crosstalk in the brain function and behavior, we further chose key metabolites with available GWAS, including propionic acid, β-hydroxybutyric acid (BHB), serotonin, GABA, trimethylamine N-oxide (TMAO), betaine, choline, and carnitine. These gut microbial metabolites play crucial roles in maintaining a healthy neuropsychiatric function, and if dysregulated, potentially causally linked to neuropsychiatric disorders according to previous studies [3, 24, 25]. We searched PubMed for GWASs of the gut metabolites and leveraged summary-level data from a recent GWAS of the human metabolome conducted among 2076 participants of the Framingham Heart Study (Table 1) [20]. Since few loci identified by gut metabolite GWAS have reached the level of genome-wide significance, we only selected SNPs at thresholds for suggestive genome-wide significance (*P* < 1 × 10^−5^) from the GWAS for each metabolite (Table S2).

#### Neuropsychiatric disorders

We searched PubMed for GWASs of the neuropsychiatric disorders and identified SNPs with genome-wide significant (*P* < 5 × 10^−8^) associations for AD [21], MDD [22], and SCZ [23], respectively (Table 1, Table S3). Summarized data for AD were obtained from the International Genomics of Alzheimer’s Project (IGAP), including 25,580 AD cases and 48,466 controls, and the analysis was adjusted for age, sex, and principal components when necessary [21]. Genetic associations for MDD were obtained from Psychiatric Genomics Consortium 29 (PGC29) including135,458 MDD cases and 344,901 controls, using sex and age as covariates [22]. Genetic associations for SCZ were obtained from a meta-analysis of Sweden and PGC including 13,833 SCZ cases and 18,310 controls [23]. Detailed information on diagnostic criteria for AD, MDD, and SCZ are provided in Table S4. These GWASs identified 19 SNPs for AD, 44 SNPs for MDD, and 24 SNPs for SCZ (*P* < 5 × 10^−8^), respectively (Table S3).

### Statistical analysis

For instrumental variables, we only selected independent genetic variants which are not in linkage disequilibrium (LD) (defined as *r*^2^ < 0.1) with other genetic variants based on European ancestry reference data from the 1000 Genomes Project. We chose the variant with the lowest *P* value for association with the exposure when genetic variants were in LD. Moreover, for SNPs that were not available in GWASs of the outcome, we used the LD proxy search on the online platform (https://snipa.helmholtz-muenchen.de/snipa3/index.php/) to replace them with the proxy SNPs identified in high-LD (*r*^2^ > 0.8) or discard them if the proxies were not available. Power calculations for the MR study were conducted based on the website: mRnd (http://cnsgenomics.com/shiny/mRnd/).

We combined MR estimates by using inverse variance weighting (IVW) as primary method. Weighted mode, weighted median, and MR-Egger methods were used as sensitivity analyses. Detailed information about the MR methods mentioned above has been explained previously [26, 27]. The MR-Egger method examined for unknown horizontal pleiotropy as indicated by a non-zero intercept value. We also applied leave-one-SNP-out approach assessing the effects of removing these SNPs from the MR analysis to rule out potential pleiotropic effects. Effect estimates are reported in beta values for the continuous outcome and ORs (95% CIs) for binary outcome. Bonferroni correction was used to adjust for multiple comparisons, giving a cutoff of *P* = 7.6 × 10^−4^ for the causal effect of gut microbiota on disorders and a cutoff of *P* = 1.7 × 10^−4^ for reverse causation.

The MR analyses were conducted in the R version 3.5.1 computing environment (http://www.r-project.org) using the TwoSampleMR package (R project for Statistical Computing). This package harmonized effect of the exposure and outcome data sets including combined information on SNPs, including phenotypes, effect alleles, effect allele frequencies, effect sizes, and standard errors for each SNP. In addition, we assumed that all alleles are presented on the forward strand in harmonization. In conclusion, the bi-directional MR results using the full set of selected SNPs.

## Results

### Associations of gut microbiota and metabolites with neuropsychiatric disorders

**Fig. 1:**
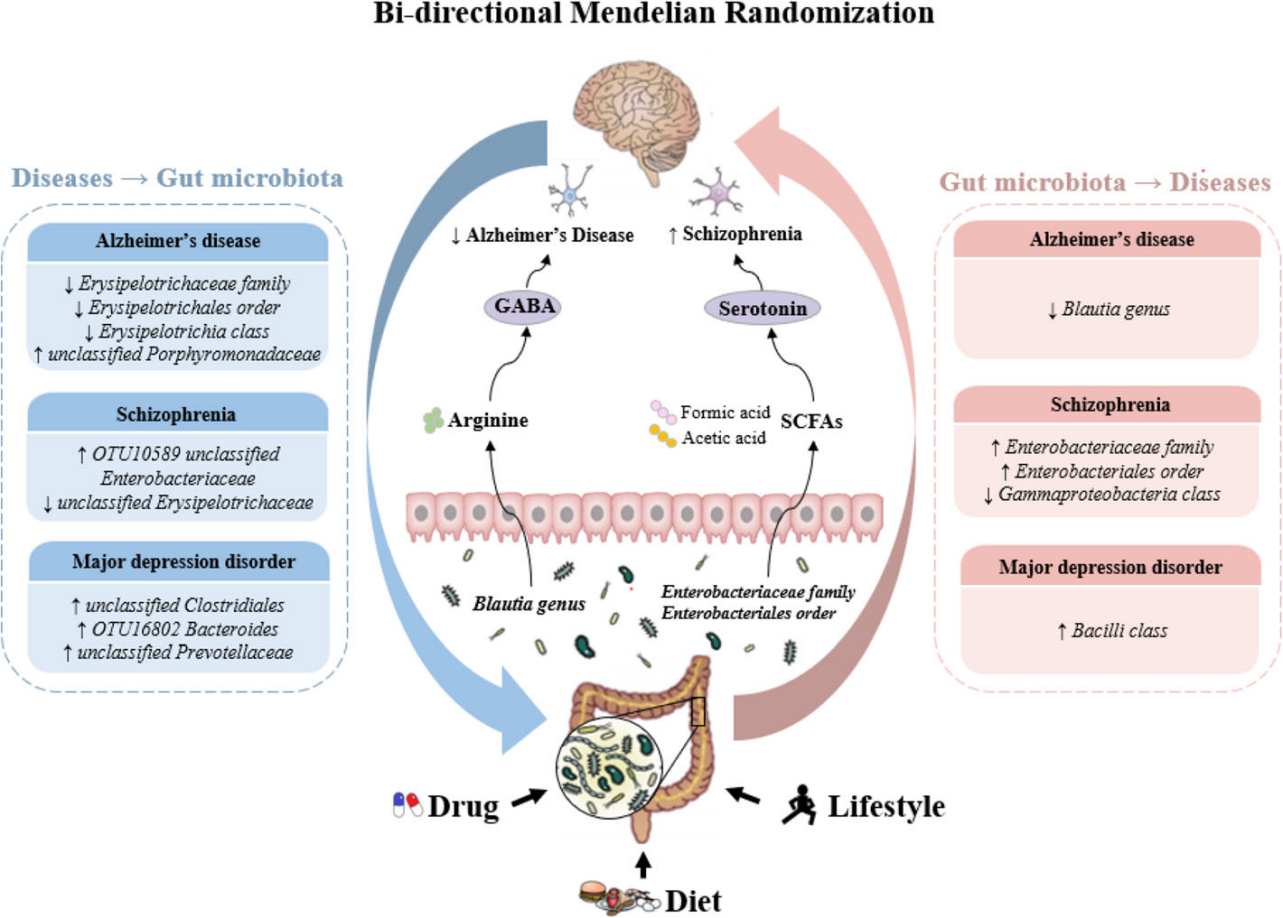
Schematic representation of the present study, highlighting for each step of the study design and the significant results obtained. We aimed to estimate causal relationships between gut microbiota (98 individual bacterial traits) and neuropsychiatric disorders (Alzheimer’s disease, major depression disorder, and schizophrenia) using a bi-directional Mendelian randomization (MR) approach (step 1). Then, we performed a two-sample MR analysis to identify which microbiota metabolites associated with these disorders (step 2). Finally, we identified 14 individual bacterial traits and 2 gut metabolites to be associated with these disorders. GABA, γ-aminobutyric acid; SCFA, short-chain fatty acids

**Fig. 2:**
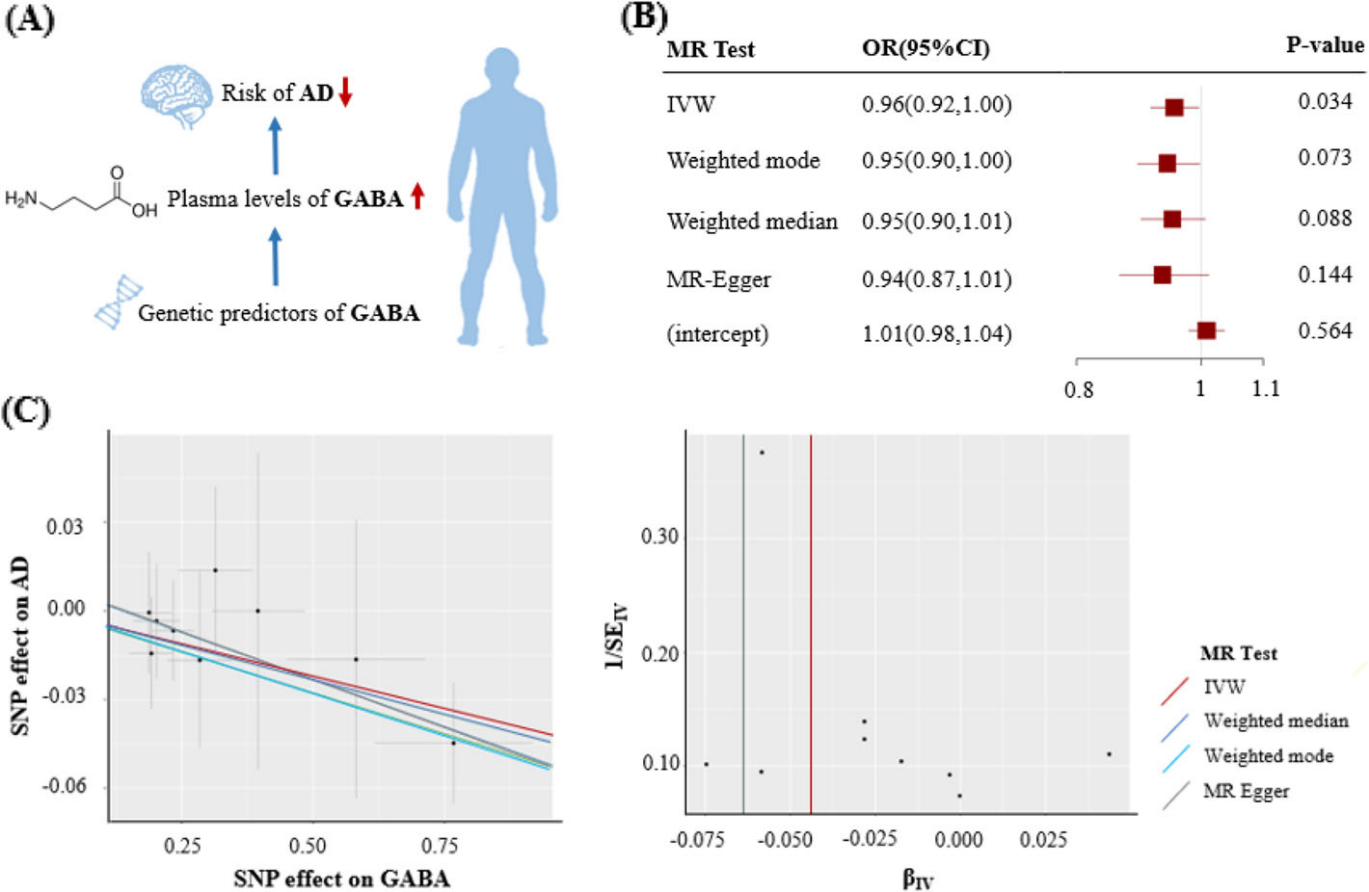
Causal effect of GABA with the risk of AD. **a** Schematic representation of the MR analysis results: genetically determined higher GABA plasma levels were potentially associated with a lower risk of AD. **b** The odds ratios (95% confidence interval) for AD per 10 units increase in GABA, as estimated in the inverse-variance weighted, weighted mode, weighted median, and MR-Egger MR analysis. The intercept of MR-Egger can be interpreted as a test of overall unbalanced horizontal pleiotropy. **c** The scatter plot represents instruments association including AD associations (*y*-axis) against instrument GABA associations (*x*-axis). The tunnel plot represents instrument precision (i.e., instrument AD regression coefficients divided by the correspondent instrument GABA SEs) (*y*-axis) against individual instrument ratio estimates in log odds ratio of AD (*x*-axis). β_IV_ indicates odds ratio estimate per 1-ln 10 units increment in GABA levels. AD, Alzheimer’s disease; OR, odds ratio; CI, confidence interval; SNP, single-nucleotide polymorphism; SE, standard error; IVW, inverse variance weighted

We found suggestive evidence of a protective effect of the host-genetic-driven increase in *Blautia* on the risk of AD (per relative abundance: OR, 0.88; 95% CI, 0.79–0.99; *P* = 0.028) (Fig. 1, Figure S1). Importantly, we further observed suggestive evidence that genetically elevated gut metabolite GABA was associated with a lower risk of AD (per 10 units: 0.96; 0.92–1.00; *P* = 0.034) (Figs. 1 and 2).

**Fig. 3:**
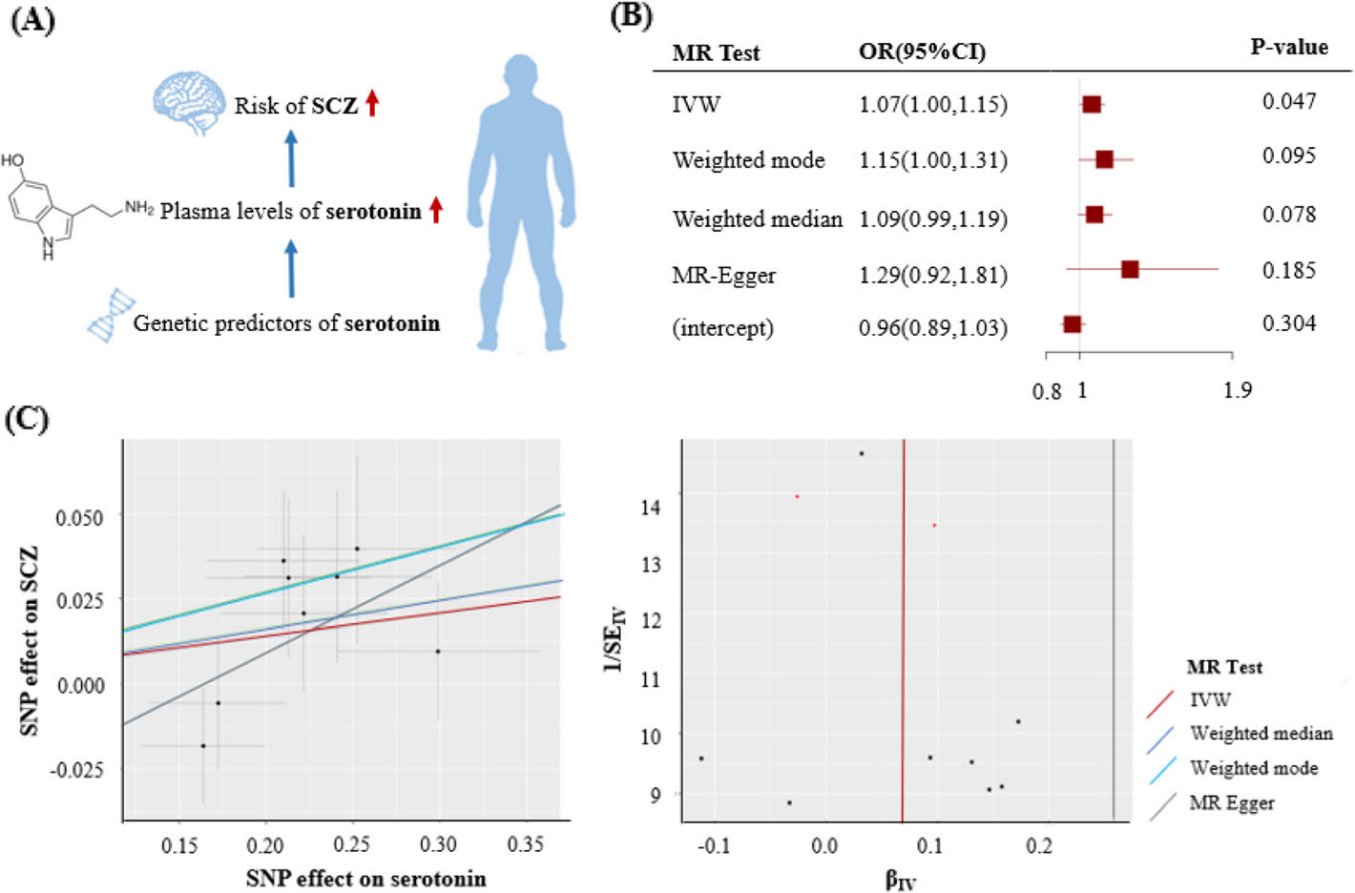
Causal effect of serotonin with the risk of SCZ. **a** Schematic representation of the MR analysis results: genetically determined higher serotonin plasma levels were potentially associated with a higher risk of SCZ. **b** The odds ratios (95% confidence interval) for SCZ per 10 units increase in serotonin, as estimated in the inverse-variance weighted, weighted mode, weighted median, and MR-Egger MR analysis. The intercept of MR-Egger can be interpreted as a test of overall unbalanced horizontal pleiotropy. **c** The scatter plot represents instruments association including SCZ associations (*y*-axis) against instrument serotonin associations (*x*-axis). The tunnel plot represents instrument precision (i.e., instrument SCZ regression coefficients divided by the correspondent instrument serotonin SEs) (*y*-axis) against individual instrument ratio estimates in log odds ratio of SCZ (*x*-axis). β_IV_ indicates odds ratio estimate per 1-ln 10 units increment in serotonin levels. SCZ, schizophrenia

Furthermore, the host-genetic-driven increases in *Enterobacteriaceae family* and *Enterobacteriales order* were potentially related to a higher risk of SCZ (1.09; 1.00–1.18; *P* = 0.048), while *Gammaproteobacteria class* was related to a lower risk of SCZ (0.90; 0.83–0.98; *P* = 0.011) (Fig. 1, Figure S1). Interestingly, gut production of serotonin was potentially associated with a higher risk of SCZ (1.07; 1.00–1.15; *P* = 0.047) (Figs. 1 and 3). In addition, we found suggestive association of the host-genetic-driven increase in *Bacilli class* with a higher risk of MDD (1.07; 1.02–1.12; *P* = 0.010) (Fig. 1, Figure S1).

Sensitivity analysis yielded similar results for the causal effects of gut microbiota on neuropsychiatric disorders, and no horizontal pleiotropy or outliers were observed (Tables S5 and S6). No significant results were found for any of other selected gut microbiota or metabolites with neuropsychiatric disorders (Table S7). MR power calculation showed strong power to detect significant (*P* < 7.6 × 10^−4^) causal effect (OR = 1.2) for most of gut microbiota with the risk of AD, MDD, and SCZ, respectively (Table S8).

### Associations of neuropsychiatric disorders with gut microbiota

In the opposite direction, we applied the MR method to investigate the causal relationship of neuropsychiatric disorders with gut microbiota. We found a suggestive association of AD with lower relative abundance of *Erysipelotrichaceae family*, *Erysipelotrichales order*, and *Erysipelotrichia class* (per 1-unit odds ratio: Beta±SE, − 0.274 ± 0.090; *P* = 0.003) and higher relative abundance of *unclassified Porphyromonadaceae* (0.351 ± 0.170; *P* = 0.040) (Fig. 1, Table S9). Additionally, MDD was associated with higher relative abundance of *unclassified Clostridiales* (0.577 ± 0.241; *P* = 0.017), *OTU16802 Bacteroides* (0.842 ± 0.386; *P* = 0.029), and *unclassified Prevotellaceae* (0.978 ± 0.464; *P* = 0.035) (Fig. 1, Table S9). We further identified that SCZ was nominally related to 2 genera, including higher relative abundance of *OTU10589 unclassified Enterobacteriaceae* (0.457 ± 0.220; *P* = 0.037) and lower relative abundance of *unclassified Erysipelotrichaceae* (− 0.248 ± -0.019; *P* = 0.045) (Fig. 1, Table S9).

Associations were almost consistent in sensitivity analyses using the weighted mode and weighted median methods. The MR-Egger method showed directional pleiotropy in the analysis of association between MDD and *OTU16802 Bacteroides* (*P* = 0.022) but not in any other potential significant associations (Table S9). However, we had limited power (all less than 50%) to test significant (*P* < 1.7 × 10^−4^) causal effect (Beta = 0.5) of the risk of AD, MDD, and SCZ on specific gut microbiota (data not shown), possibly due to small sample size of the gut microbiota GWAS.

## Discussion

In this two-sample bi-directional MR study, we found suggestive evidence of causal relationships of *Blautia* with AD, of *Enterobacteriaceae family*, *Enterobacteriales order,* and *Gammaproteobacteria class* with SCZ, and of *Bacilli class* with MDD. More importantly, several neurotransmitters such as GABA and serotonin produced by gut microbiota were also potentially linked to the risks of neuropsychiatric disorders, implying their important roles in microbiota-host crosstalk in the brain function and behavior. In the other direction, our results suggested that neuropsychiatric disorders, including AD, SCZ, and MDD might alter the composition of gut microbiota.

Microbiota-gut-brain communication has been shown to play a key role in cognitive function [2]. However, animal studies regarding the effects of *Blautia genus* on AD have yielded conflicting results, but extrapolating these findings to human beings is challenging [28, 29]. A cohort study (*n* = 108) reported that decreased proportion of *Blautia hansenii* was associated with a higher risk of AD [30], while two case-control studies observed that *Blautia* were more abundant in AD patients [5, 31]. Although the direction of associations between *Blautia* and the risk of AD substantially differed across studies, one consistent finding was that gut microbial neurotransmitter GABA, a downstream product of *Blautia*-dependent arginine metabolism, was related to a reduced risk of AD. Notably, lower levels of gut product of GABA were observed in patients with AD in several case-control studies [32, 33]. In this bi-directional MR study, our results for the first time provide evidence of a causal relationship between relative abundance of *Blautia* and AD. More importantly, we demonstrated that elevated GABA was potentially associated with a lower risk of AD. Our findings supported previous meta-analysis of 35 observational studies which suggested that GABA level in AD were significantly lower than that of controls [12]. Our findings suggest that GABA produced by gut microbiota may play an important role in microbiota-host crosstalk in the brain function and behavior. Although not significant, our findings show very similar association directions for *Blautia* with MDD and SCZ. Our findings are in line with recent studies which indicated that decreased *Blautia* was associated with an increased risk of autistic spectrum disorder (ASD), suggesting a general change associated with psychiatric disorders [34].

There are many potential pathways linking specific gut microbiota to AD, among which metabolites produced by gut microbiota may play an important role. It is worth noting that GABA, as a primary inhibitory neurotransmitter in the human central nervous system (CNS), has been shown to shape neurological processes and cognition [35]. Recent evidence has demonstrated that GABAergic functions could be an essential factor in the whole stage of AD pathogenesis which seemed to be more resistant to neurodegenerative changes in aged brain [36, 37]. Our MR results that increased GABA levels was potentially associated with a lower risk of AD lent further support to the hypotheses. The biological mechanisms of GABA production include degradation of putrescine, decarboxylation of glutamate, or from arginine or ornithine [8]. Interestingly, the genus *Blautia* has shown a strong correlation with arginine metabolism [38], which may be involved in AD pathogenesis by regulating its downstream products such as GABA, supporting the potential pathway [39]. Since AD does not break out suddenly but develops through a long prodromal phase instead, it is plausible that our findings may be potentially effective in early interventions of such disorder in the future by targeting the microbiota (e.g., gut microbiota transplantation, psychobiotics, or antibiotics).

Recently, *Enterobacteriales family* and *Gammaproteobacteria class* have been identified to be important biomarkers of SCZ in recent cross-sectional studies, consistent with our findings [6, 40]. Furthermore, a case-control study (*n* = 364) identified a strong relationship of lower platelet serotonin concentrations with depressive symptoms of SCZ [14]. However, available evidence is still largely inadequate since observational studies mainly rely on self-reported information and are susceptible to confounding (e.g., diet and health status) and reverse causation bias. Ertugrul et al. observed plasma serotonin increased while platelet serotonin decreased in SCZ patients after clinical treatments, which was inconsistent with our findings [13]. In addition, our results support the finding that increased *Bacilli* is potentially associated with a higher risk of MDD, possibly involving dopamine metabolism which might play a role in the major symptoms of MDD [41, 42]. A meta-analysis of RCTs showed that probiotics, typically including *Lactobacillus* and *Bifidobacterium*, had some benefit for MDD, but we found no associations for these microbiota, possibly due to the synergistic effect of gut microbiome so that the influence of a particular taxon may be different from multiple taxa [43]. Furthermore, these clinical trials might draw biased conclusions because of small sample sizes (ranging from 17 to 110) or short-term effects (ranging from 3 to 24 weeks). Therefore, a large and long-term RCT in a well-characterized population using probiotic capsules containing specific microbiota might provide further evidence for the gut-brain axis in these disorders. Importantly, epidemiological study indicated that elevated *Enterobacteriales* was also associated with a higher risk of ASD, suggesting that the same changes in intestinal microbiota composition might lead to different outcomes due to gene-gene interactions and gene-environment interactions [44]. Although our results showed no significant association for *Gammaproteobacteria* and MDD, animal models found increased levels of *Gammaproteobacteria* were also associated with higher MDD risk and fluoxetine treatment was effective, implying strong correlations between gut microbiota and anxiety- and depression-like behaviors [45].

The serotonin hypothesis of SCZ originated from earlier studies of interactions between the hallucinogenic drug D-lysergic acid diethylamide and serotonin in peripheral systems. However, direct evidence of serotonergic dysfunction in the pathogenesis of SCZ remains unclear [46]. According to the principle of brain plasticity, glutamate signals are destroyed by serotonergic overdrive, leading to neuronal hypometabolism, synaptic atrophy, and gray matter loss in the end [47]. Our findings that genetically increased serotonin levels was potentially related to a high risk of SCZ using a MR approach supported such hypothesis. Importantly, *Enterobacteriaceae family* and *Enterobacteriales order* can produce SCFAs (e.g., acetic acid and formic acid) in carbohydrate fermentation, thus inducing serotonin biosynthesis by enterochromaffin cells which are the major producers of serotonin, and ultimately increasing the risk of SCZ [48, 49]. Our novel findings highlighted the potentially important role of gut microbiota-related neurotransmitters in effective and benign therapies of psychiatric disorders.

Furthermore, we also found that neuropsychiatric disorders might alter the composition of gut microbiota. Our findings were consistent with a small case-control study (*n* = 50) suggesting that *Erysipelotrichaceae family* were all less abundant in patients with AD [5]. An observational study showed that *Porphyromonadaceae* were associated with poor cognitive performance, partly consistent with our results [50]. However, the results from animal studies are conflicting. Although several animal studies suggested that anti-AD microbes, such as *Erysipelotrichiaceae*, decreased in mouse models with AD, and *Porphyromonadaceae* increased in aged mice [28, 51], other animal studies showed that the relative abundance of *Erysipelotrichiaceae* was positively correlated with AD [52, 53]. Therefore, the association of neuropsychiatric disorders with specific gut microbiota requires further study. It is universally accepted that the CNS modulates gut microbiota compositions mainly through hypothalamic-pituitary-adrenal (HPA) axis, or classical neurotransmitters liberated by neuronal efferent activation, which explains the microbiota-host crosstalk in neuropsychiatric disorders from another direction [54].

Additionally, it is plausible that alterations in gut microbiota and related metabolites would lead to a systemic change in inflammation that may contribute to the neuroinflammation in AD, MDD, and SCZ. Increasing evidence suggests that bacteria populating the gut microbiome may excrete large quantities of lipopolysaccharides and amyloids, resulting in the pathogenesis of AD during aging when the permeability of gastrointestinal tract epithelium or blood-brain barrier increases [55]. Recent research has indicated that gut inflammation can induce activation of microglia and the kynurenine pathway, which activate systemic inflammation-inducing depressive or schizophrenic symptoms [56, 57]. Therefore, more studies are required to explore the mechanisms underlying the relationships of inflammation with the gut microbiota-brain axis and its relations with AD, MDD and SCZ.

Strengths of the present study included the bi-directional MR design and the use of summary-level data from thus far the largest GWASs. This design generally avoided bias due to reverse causation and confounding to obtain accurate results under MR assumptions. In addition, consistent results from several sensitivity analyses including the use of weighted mode, weighted median, and MR-egger methods indicate robustness of our findings. However, several limitations merit consideration. First, our results did not survive a strict Bonferroni correction adjusting for multiple comparisons, whereas as a hypothesis-driven approach, the MR study with some biological evidence was used to test epidemiologically established associations, regardless of Bonferroni corrected *P* values. Second, we used limited number of gut microbiota SNPs as instrumental variables; we cannot exclude that our findings might have been affected by weak instrument bias, although all genetic instruments were associated with the exposure (*F*-statistic > 10). Third, statistical power was limited for associations of neuropsychiatric disorders with gut microbiota, so we cannot exclude type II error as an explanation for the null results completely. Larger GWASs of gut microbiota are required to provide sufficient statistical power. However, the power was strong enough for the effect of gut microbiota on these disorders, which was our main findings in the present study. Fourth, our results were restricted to European ancestry. Replication with functionally relevant genetic prediction of gut microbiota is warranted given the substantial difference in gut microbiota composition among different populations. Fifth, the 16S rRNA gene sequencing only permit resolution from the genus to the phylum level rather than at a more specific level, resulting in biased results if some specific species contributed to neuropsychiatric disorders. Finally, gut microbiota might be influenced by environmental factors such as dietary habits or health status, which led to lower variance explained by genetic instruments. However, we could not test whether genetic instruments are associated with these confounders such as diet or lifestyle information in the present study where such information is not available.

## Conclusions

In summary, our findings supported several potential associations between specific gut microbiota and neuropsychiatric disorders and highlighted the important roles of microbial neurotransmitters such as GABA and serotonin in microbiota-host crosstalk in neuropsychiatric disorders. Further investigations in understanding the underlying mechanisms of gut microbiota in the development of neuropsychiatric disorders are required.

## Supplementary information

**Additional file 1: Figure S1.** Odds ratio for association of genetically predicted gut microbiota with neuropsychological diseases. **Table S1.** Characteristics of selected SNPs for core gut microbiota. **Table S2.** Characteristics of selected SNPs for gut metabolites. **Table S3.** Characteristics of selected SNPs for neuropsychological diseases. **Table S4.** Description of the diagnostic assessment for neuropsychological diseases. **Table S5.** Associations between genetically predicted gut microbiota and neuropsychological diseases in sensitivity analyses. **Table S6.** Associations between genetically predicted gut microbiota and neuropsychological diseases in a leave-one-out approach. **Table S7.** Associations between genetically predicted metabolites and neuropsychological diseases using IVW method. **Table S8.** MR Power calculation for detecting significant (*P* < 7.6 × 10-4) causal effect (OR = 1.2) of gut microbiome on the risk of AD, MDD, and SCZ. **Table S9.** Effect estimates for association of genetically predicted neuropsychological diseases with gut microbiota using four Mendelian randomization methods.

## Data Availability

All data used in the present study were obtained from genome-wide association study summary statistics which were publicly released by genetic consortia.

## Abbreviations

AD: Alzheimer’s disease
MDD: Major depressive disorder
SCZ: Schizophrenia
MR: Mendelian randomization
GWAS: Genome-wide association study
GABA: γ-Aminobutyric acid
SCFA: Short-chain fatty acid
BHB: β-Hydroxybutyric acid
TMAO: Trimethylamine N-oxide
SNP: Single nucleotide polymorphism
IGAP: International Genomics of Alzheimer’s Project
PGC29: Psychiatric Genomics Consortium 29
LD: Linkage disequilibrium
IVW: Inverse variance weighting
CNS: Central nervous system
HPA: Hypothalamic-pituitary-adrenal
ASD: Autistic spectrum disorder

## References

[1] Valles-Colomer M, Falony G, Darzi Y, Tigchelaar EF, Wang J, Tito RY, Schiweck C, Kurilshikov A, Joossens M, Wijmenga C (2019). The neuroactive potential of the human gut microbiota in quality of life and depression. Nat Microbiol.

[2] Fung TC, Olson CA, Hsiao EY (2017). Interactions between the microbiota, immune and nervous systems in health and disease. Nat Neurosci.

[3] Caspani G, Swann J (2019). Small talk: microbial metabolites involved in the signaling from microbiota to brain. Curr Opin Pharmacol.

[4] van de Wouw M, Boehme M, Lyte JM, Wiley N, Strain C, O'Sullivan O, Clarke G, Stanton C, Dinan TG, Cryan JF (2018). Short-chain fatty acids: microbial metabolites that alleviate stress-induced brain-gut axis alterations. J Physiol.

[5] Vogt NM, Kerby RL, Dill-McFarland KA, Harding SJ, Merluzzi AP, Johnson SC, Carlsson CM, Asthana S, Zetterberg H, Blennow K (2017). Gut microbiome alterations in Alzheimer’s disease. Sci Rep.

[6] Shen Y, Xu J, Li Z, Huang Y, Yuan Y, Wang J, Zhang M, Hu S, Liang Y (2018). Analysis of gut microbiota diversity and auxiliary diagnosis as a biomarker in patients with schizophrenia: a cross-sectional study. Schizophr Res.

[7] Naseribafrouei A, Hestad K, Avershina E, Sekelja M, Linlokken A, Wilson R, Rudi K (2014). Correlation between the human fecal microbiota and depression. Neurogastroenterol Motil.

[8] Strandwitz P, Kim KH, Terekhova D, Liu JK, Sharma A, Levering J, McDonald D, Dietrich D, Ramadhar TR, Lekbua A (2019). GABA-modulating bacteria of the human gut microbiota. Nat Microbiol.

[9] Strandwitz P (2018). Neurotransmitter modulation by the gut microbiota. Brain Res.

[10] Huang D, Liu D, Yin J, Qian T, Shrestha S, Ni H (2017). Glutamate-glutamine and GABA in brain of normal aged and patients with cognitive impairment. Eur Radiol.

[11] Peitl V, Štefanović M, Karlović D (2017). Depressive symptoms in schizophrenia and dopamine and serotonin gene polymorphisms. Prog Neuropsychopharmacol Biol Psychiatry.

[12] Manyevitch R, Protas M, Scarpiello S, Deliso M, Bass B, Nanajian A, Chang M, Thompson SM, Khoury N, Gonnella R (2018). Evaluation of metabolic and synaptic dysfunction hypotheses of Alzheimer’s disease (AD): a meta-analysis of CSF markers. Curr Alzheimer Res.

[13] Ertugrul A, Ucar G, Basar K, Demir B, Yabanoglu S, Ulug B (2007). Influence of clozapine on platelet serotonin, monoamine oxidase and plasma serotonin levels. Psychiatry Res.

[14] Peitl V, Vidrih B, Karlović Z, Getaldić B, Peitl M, Karlović D (2016). Platelet serotonin concentration and depressive symptoms in patients with schizophrenia. Psychiatry Res.

[15] Emdin CA, Khera AV, Kathiresan S (2017). Mendelian randomization. JAMA.

[16] Sanna S, van Zuydam NR, Mahajan A, Kurilshikov A, Vich Vila A, Võsa U, Mujagic Z, Masclee AAM, Jonkers DMAE, Oosting M (2019). Causal relationships among the gut microbiome, short-chain fatty acids and metabolic diseases. Nat Genet.

[17] Turpin W, Espin-Garcia O, Xu W, Silverberg MS, Kevans D, Smith MI, Guttman DS, Griffiths A, Panaccione R, Otley A (2016). Association of host genome with intestinal microbial composition in a large healthy cohort. Nat Genet.

[18] Goodrich JK, Davenport ER, Beaumont M, Jackson MA, Knight R, Ober C, Spector TD, Bell JT, Clark AG, Ley RE (2016). Genetic determinants of the gut microbiome in UK twins. Cell Host Microbe.

[19] Wang J, Thingholm LB, Skiecevičienė J, Rausch P, Kummen M, Hov JR, Degenhardt F, Heinsen F-A, Rühlemann MC, Szymczak S (2016). Genome-wide association analysis identifies variation in vitamin D receptor and other host factors influencing the gut microbiota. Nat Genet.

[20] Rhee EP, Ho JE, Chen MH, Shen D, Cheng S, Larson MG, Ghorbani A, Shi X, Helenius IT, O'Donnell CJ (2013). A genome-wide association study of the human metabolome in a community-based cohort. Cell Metab.

[21] Lambert JC, Ibrahim-Verbaas CA, Harold D, Naj AC, Sims R, Bellenguez C, DeStafano AL, Bis JC, Beecham GW, Grenier-Boley B (2013). Meta-analysis of 74,046 individuals identifies 11 new susceptibility loci for Alzheimer’s disease. Nat Genet.

[22] Wray NR, Ripke S, Mattheisen M, Trzaskowski M, Byrne EM, Abdellaoui A, Adams MJ, Agerbo E, Air TM, Andlauer TMF (2018). Genome-wide association analyses identify 44 risk variants and refine the genetic architecture of major depression. Nat Genet.

[23] Ripke S, O'Dushlaine C, Chambert K, Moran JL, Kähler AK, Akterin S, Bergen SE, Collins AL, Crowley JJ, Fromer M (2013). Genome-wide association analysis identifies 13 new risk loci for schizophrenia. Nat Genet.

[24] Sabokdast M, Habibi-Rezaei M, Moosavi-Movahedi AA, Ferdousi M, Azimzadeh-Irani E, Poursasan N (2015). Protection by beta-Hydroxybutyric acid against insulin glycation, lipid peroxidation and microglial cell apoptosis. Daru.

[25] Zalar B, Haslberger A, Peterlin B (2018). The role of microbiota in depression - a brief review. Psychiatr Danub.

[26] Bowden J, Davey Smith G, Haycock PC, Burgess S (2016). Consistent estimation in Mendelian randomization with some invalid instruments using a weighted median estimator. Genet Epidemiol.

[27] Hartwig FP, Davey Smith G, Bowden J (2017). Robust inference in summary data Mendelian randomization via the zero modal pleiotropy assumption. Int J Epidemiol.

[28] Xin Y, Diling C, Jian Y, Ting L, Guoyan H, Hualun L, Xiaocui T, Guoxiao L, Ou S, Chaoqun Z (2018). Effects of oligosaccharides from Morinda officinalis on gut microbiota and metabolome of APP/PS1 transgenic mice. Front Neurol.

[29] Park J-Y, Choi J, Lee Y, Lee J-E, Lee E-H, Kwon H-J, Yang J, Jeong B-R, Kim Y-K, Han P-L (2017). Metagenome analysis of bodily microbiota in a mouse model of Alzheimer disease using bacteria-derived membrane vesicles in blood. Exp Neurobiol.

[30] Haran JP, Bhattarai SK, Foley SE, Dutta P, Ward DV, Bucci V, McCormick BA (2019). Alzheimer’s disease microbiome is associated with dysregulation of the anti-inflammatory P-glycoprotein pathway. mBio.

[31] Li B, He Y, Ma J, Huang P, Du J, Cao L, Wang Y, Xiao Q, Tang H, Chen S (2019). Mild cognitive impairment has similar alterations as Alzheimer’s disease in gut microbiota. Alzheimers Dement.

[32] Gueli MC, Taibi G (2013). Alzheimer’s disease: amino acid levels and brain metabolic status. Neurol Sci.

[33] Jiménez-Jiménez FJ, Molina JA, Gómez P, Vargas C, de Bustos F, Benito-León J, Tallón-Barranco A, Ortí-Pareja M, Gasalla T, Arenas J (1998). Neurotransmitter amino acids in cerebrospinal fluid of patients with Alzheimer’s disease. J Neural Transm (Vienna).

[34] Liu F, Li J, Wu F, Zheng H, Peng Q, Zhou H (2019). Altered composition and function of intestinal microbiota in autism spectrum disorders: a systematic review. Transl Psychiatry.

[35] de JR De-Paula V, Forlenza AS, Forlenza OV (2018). Relevance of gutmicrobiota in cognition, behaviour and Alzheimer’s disease. Pharmacol Res.

[36] Rissman RA, De Blas AL, Armstrong DM (2007). GABA(A) receptors in aging and Alzheimer’s disease. J Neurochem.

[37] Govindpani K, Calvo-Flores Guzmán B, Vinnakota C, Waldvogel HJ, Faull RL, Kwakowsky A (2017). Towards a better understanding of GABAergic remodeling in Alzheimer’s disease. Int J Mol Sci.

[38] Liu X, Zheng H, Lu R, Huang H, Zhu H, Yin C, Mo Y, Wu J, Liu X, Deng M (2019). Intervening effects of total alkaloids of Corydalis saxicola Bunting on rats with antibiotic-induced gut microbiota dysbiosis based on 16S rRNA gene sequencing and untargeted metabolomics analyses. Front Microbiol.

[39] Bergin DH, Jing Y, Zhang H, Liu P (2015). A single intracerebroventricular Aβ25-35 infusion leads to prolonged alterations in arginine metabolism in the rat hippocampus and prefrontal cortex. Neuroscience.

[40] Nguyen TT, Kosciolek T, Maldonado Y, Daly RE, Martin AS, McDonald D, Knight R, Jeste DV (2019). Differences in gut microbiome composition between persons with chronic schizophrenia and healthy comparison subjects. Schizophr Res.

[41] Cao G, Tao F, Hu Y, Li Z, Zhang Y, Deng B, Zhan X (2019). Positive effects of a Clostridium butyricum-based compound probiotic on growth performance, immune responses, intestinal morphology, hypothalamic neurotransmitters, and colonic microbiota in weaned piglets. Food Funct.

[42] Opmeer EM, Kortekaas R, Aleman A (2010). Depression and the role of genes involved in dopamine metabolism and signalling. Prog Neurobiol.

[43] Barbosa RSD, Vieira-Coelho MA. Probiotics and prebiotics: focus on psychiatric disorders - a systematic review. Nutr Rev. 2020;78(6):437-50. 10.1093/nutrit/nuz080 31769847

[44] Liu S, Li E, Sun Z, Fu D, Duan G, Jiang M, Yu Y, Mei L, Yang P, Tang Y (2019). Altered gut microbiota and short chain fatty acids in Chinese children with autism spectrum disorder. Sci Rep.

[45] Sun L, Zhang H, Cao Y, Wang C, Zhao C, Wang H, Cui G, Wang M, Pan Y, Shi Y (2019). Fluoxetine ameliorates dysbiosis in a depression model induced by chronic unpredicted mild stress in mice. Int J Med Sci.

[46] Yang AC, Tsai S-J (2017). New targets for schizophrenia treatment beyond the dopamine hypothesis. Int J Mol Sci.

[47] Abi-Dargham A, Laruelle M, Aghajanian GK, Charney D, Krystal J (1997). The role of serotonin in the pathophysiology and treatment of schizophrenia. J Neuropsychiatry Clin Neurosci.

[48] Ge X, Pan J, Liu Y, Wang H, Zhou W, Wang X (2018). Intestinal crosstalk between microbiota and serotonin and its impact on gut motility. Curr Pharm Biotechnol.

[49] Blander JM, Longman RS, Iliev ID, Sonnenberg GF, Artis D (2017). Regulation of inflammation by microbiota interactions with the host. Nat Immunol.

[50] Bajaj JS, Ahluwalia V, Steinberg JL, Hobgood S, Boling PA, Godschalk M, Habib S, White MB, Fagan A, Gavis EA (2016). Elderly patients have an altered gut-brain axis regardless of the presence of cirrhosis. Sci Rep.

[51] Du C-T, Gao W, Ma K, Yu S-X, Li N, Yan S-Q, Zhou F-H, Liu Z-Z, Chen W, Lei L-C (2018). MicroRNA-146a deficiency protects against Listeria monocytogenes infection by modulating the gut microbiota. Int J Mol Sci.

[52] Harach T, Marungruang N, Duthilleul N, Cheatham V, Mc Coy KD, Frisoni G, Neher JJ, Fak F, Jucker M, Lasser T (2017). Reduction of Abeta amyloid pathology in APPPS1 transgenic mice in the absence of gut microbiota. Sci Rep.

[53] Bauerl C, Collado MC, Diaz Cuevas A, Vina J, Perez Martinez G (2018). Shifts in gut microbiota composition in an APP/PSS1 transgenic mouse model of Alzheimer’s disease during lifespan. Lett Appl Microbiol.

[54] Montiel-Castro AJ, González-Cervantes RM, Bravo-Ruiseco G, Pacheco-López G (2013). The microbiota-gut-brain axis: neurobehavioral correlates, health and sociality. Front Integr Neurosci.

[55] Lin L, Zheng LJ, Zhang LJ (2018). Neuroinflammation, gut microbiome, and Alzheimer’s disease. Mol Neurobiol.

[56] Simkin DR (2019). Microbiome and mental health, specifically as it relates to adolescents. Curr Psychiatry Rep.

[57] Inta D, Lang UE, Borgwardt S, Meyer-Lindenberg A, Gass P (2017). Microglia activation and schizophrenia: lessons from the effects of minocycline on postnatal neurogenesis, neuronal survival and synaptic pruning. Schizophr Bull.

