# Supplementary material for: Associations between gut microbiota and Alzheimer’s disease, major depressive disorder, and schizophrenia
Source: J Neuroinflammation. 2020 Oct 2;17:288. doi: 10.1186/s12974-020-01961-8 (PMC7532639; doi:10.1186/s12974-020-01961-8)
Supplement: Supplementary file 1 — Additional file 1: Figure S1. Odds ratio for association of genetically predicted gut microbiota with neuropsychological diseases. Table S1. Characteristics of selected SNPs for core gut microbiota. Table S2. Characteristics of selected SNPs for gut metabolites. Table S3. Characteristics of selected SNPs for neuropsychological diseases. Table S4. Description of the diagnostic assessment for neuropsychological diseases. Table S5. Associations between genetically predicted gut microbiota and neuropsychological diseases in sensitivity analyses. Table S6. Associations between genetically predicted gut microbiota and neuropsychological diseases in a leave-one-out approach. Table S7. Associations between genetically predicted metabolites and neuropsychological diseases using IVW method. Table S8. MR Power calculation for detecting significant (P < 7.6 × 10^-4^) causal effect (OR = 1.2) of gut microbiome on the risk of AD, MDD, and SCZ. Table S9. Effect estimates for association of genetically predicted neuropsychological diseases with gut microbiota using four Mendelian randomization methods. [file 12974_2020_1961_MOESM1_ESM.docx]

**Additional File 1**

**Figure S1.** Odds ratio for association of genetically predicted gut microbiota with neuropsychological diseases.

**Table S1.** Characteristics of selected SNPs for core gut microbiota.

**Table S2.** Characteristics of selected SNPs for gut metabolites.

**Table S3.** Characteristics of selected SNPs for neuropsychological diseases.

**Table S4.** Description of the diagnostic assessment for neuropsychological diseases.

**Table S5.** Associations between genetically predicted gut microbiota and neuropsychological diseases in sensitivity analyses.

**Table S6.**Associations between genetically predicted gut microbiota and neuropsychological diseases in a leave-one-out approach.

**Table S7.** Associations between genetically predicted metabolites and neuropsychological diseases using IVW method.

**Table S8.** MR Power calculation for detecting significant (P < 7.6×10-4) causal effect (OR = 1.2) of gut microbiome on the risk of AD, MDD, and SCZ.

**Table S9.** Effect estimates for association of genetically predicted neuropsychological diseases with gut microbiota using four Mendelian randomization methods.

**Figure S1. Odds ratio for association of genetically predicted gut microbiota with neuropsychological diseases.**


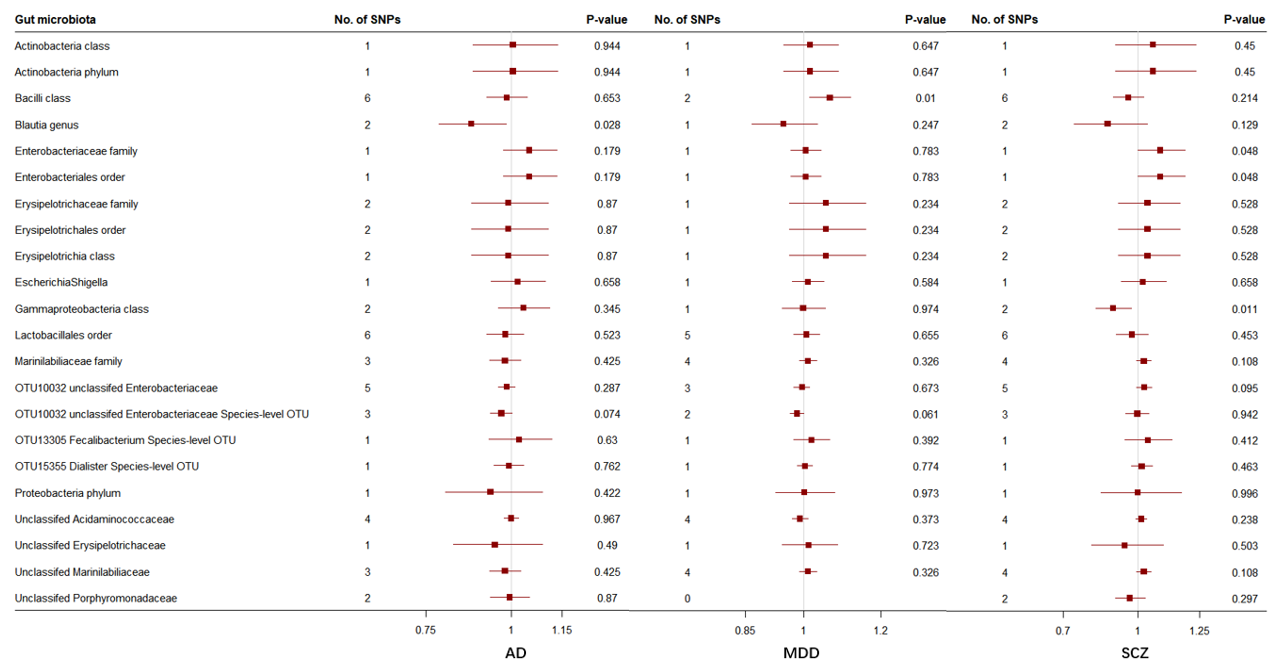


OR: odds ratio; CI: confidence internal; SNP, single-nucleotide polymorphism; AD, Alzheimer’s disease; MDD, major depressive; SCZ, schizophrenia. OR (95% CI) means risk of amyotrophic neuropsychological diseases per 1-allele increase in single nucleotide polymorphisms related to greater abundance of gut microbiota.

**Table S1. Characteristics of selected SNPs for core gut microbiota.**

| **Core gut microbiota** | **SNP** | **Chr.** | **Locus start** | **Locus end** | **A1** | **A2** | **P** | **Beta** | **SE** | β**-div *P*** | **Nearest gene** | **Genes in locus** | **Variance explained** |
| --- | --- | --- | --- | --- | --- | --- | --- | --- | --- | --- | --- | --- | --- |
| *Actinobacteria class* | rs34613612 | 21 | 32,184,901 | 32,204,347 | C | G | 6.34E-10 | 0.25 | 0.04 | 9.87E-03 | KRTAP8-1 | KRTAP8-1 | 0.0209 |
| *Actinobacteria phylum* | rs34613612 | 21 | 32,184,901 | 32,204,347 | C | G | 6.34E-10 | 0.25 | 0.04 | 9.87E-03 | KRTAP8-1 | KRTAP8-1 | 0.0209 |
| *Bacilli class* | rs10928827 | 2 | 129,426,740 | 129,473,850 | G | A | 1.02E-08 | -0.22 | 0.04 | 0.19 | HS6ST1 | - | 0.0180 |
| *Bacilli class* | rs148330122 | 19 | 38,497,288 | 38,631,252 | C | T | 1.32E-09 | -0.48 | 0.08 | 0.18 | SIPA1L3 | SIPA1L3 | 0.0201 |
| *Bacilli class* | rs2071199 | 20 | 43,030,809 | 43,037,422 |  | C | 1.24E-08 | -0.32 | 0.06 | 0.58 | HNF4A–AS1 | HNF4A | 0.0178 |
| *Bacilli class* | rs479105 | 12 | 3,357,596 | 3,393,503 | T | C | 1.21E-08 | -0.22 | 0.04 | 0.48 | PRMT8 | - | 0.0178 |
| *Bacilli class* | rs7083345 | 10 | 7,020,329 | 7,044,987 | T | C | 3.38E-10 | 0.25 | 0.04 | 0.02 | RP11-554I8.2 | - | 0.0209 |
| *Bacilli class* | rs7646786 | 3 | 185,729,634 | 185,742,372 | T | C | 2.29E-08 | -0.22 | 0.04 | 0.5 | LOC344887 | - | 0.0171 |
| *Blautia genus* | rs4669413 | 2 | 9,801,744 | 9,818,596 | T | C | 1.20E-08 | -0.18 | 0.03 | 0.75 | RP11–521D12.1 | - | 0.0178 |
| *Blautia genus* | rs79387448 | 2 | 103,099,953 | 103,239,356 | C | T | 7.68E-11 | -0.31 | 0.05 | 0.66 | SLC9A2 | SLC9A2 | 0.0231 |
| *Enterobacteriaceae family* | rs35275482 | 15 | 60,027,987 | 60,128,040 | C | A | 3.72E-11 | -0.54 | 0.08 | 0.06 | BNIP2 | - | 0.0239 |
| *Enterobacteriales order* | rs35275482 | 15 | 60,027,987 | 60,128,040 | C | A | 3.72E-11 | -0.54 | 0.08 | 0.06 | BNIP3 | - | 0.0239 |
| *Erysipelotrichaceae family* | rs11877825 | 18 | 10,566,345 | 10,595,758 | G | T | 2.82E-11 | -0.27 | 0.04 | 0.34 | NAPG | - | 0.0242 |
| *Erysipelotrichaceae family* | rs17421787 | 4 | 131,293,675 | 131,512,291 | C | G | 3.60E-08 | -0.3 | 0.05 | 0.16 | RP11-22J15.1 | - | 0.0166 |
| *Erysipelotrichales order* | rs11877825 | 18 | 10,566,345 | 10,595,758 | G | T | 2.82E-11 | -0.27 | 0.04 | 0.34 | NAPG | - | 0.0242 |
| *Erysipelotrichales order* | rs17421787 | 4 | 131,293,675 | 131,512,291 | C | G | 3.60E-08 | -0.3 | 0.05 | 0.16 | RP11-22J15.2 | - | 0.0166 |
| *Erysipelotrichia class* | rs11877825 | 18 | 10,566,345 | 10,595,758 | G | T | 2.82E-11 | -0.27 | 0.04 | 0.34 | NAPG | - | 0.0242 |
| *Erysipelotrichia class* | rs17421787 | 4 | 131,293,675 | 131,512,291 | C | G | 3.60E-08 | -0.3 | 0.05 | 0.16 | RP11-22J15.3 | - | 0.0166 |
| *EscherichiaShigella* | rs13096731 | 3 | 58,014,818 | 58,089,851 | A | G | 2.55E-08 | -0.43 | 0.08 | 0.12 | FLNB | FLNB | 0.0170 |
| *Gammaproteobacteria class* | rs4621152 | 2 | 217,857,450 | 217,924,261 | C | T | 1.40E-08 | -0.29 | 0.05 | 0.79 | AC007557.1 | - | 0.0176 |
| *Gammaproteobacteria class* | rs9300430 | 13 | 98,269,478 | 98,306,405 | C | T | 1.30E-09 | -0.61 | 0.10 | 0.12 | RAP2A | - | 0.0201 |
| *Lactobacillales order* | rs10928827 | 2 | 129,426,740 | 129,473,850 | G | A | 4.19E-09 | -0.23 | 0.04 | 0.19 | HS6ST2 | - | 0.0189 |
| *Lactobacillales order* | rs1362404 | 16 | 51,955,443 | 52,017,380 | T | G | 1.56E-08 | 0.23 | 0.04 | 7.50E-05 | TOX3 | - | 0.0175 |
| *Lactobacillales order* | rs59042687 | 3 | 95,359,287 | 95,823,523 | T | G | 6.22E-09 | -0.23 | 0.04 | 0.02 | LINC00879 | - | 0.0185 |
| *Lactobacillales order* | rs62295801 | 3 | 162,444,724 | 163,236,170 | G | T | 5.32E-10 | -0.27 | 0.04 | 0.21 | LINC01192 | LINC01192 | 0.0211 |
| *Lactobacillales order* | rs7083345 | 10 | 7,020,329 | 7,044,987 | T | C | 2.89E-09 | 0.24 | 0.04 | 0.02 | RP11-554I8.2 | - | 0.0199 |
| *Lactobacillales order* | rs7113056 | 11 | 122,091,502 | 122,154,110 | C | T | 1.72E-13 | -0.5 | 0.07 | 0.07 | RP11-166D19.1 | - | 0.0296 |
| *Marinilabiliaceae family* | rs11724031 | 4 | 77,441,448 | 77,467,405 | G | A | 2.44E-10 | -0.97 | 0.15 | 0.68 | SHROOM3 | SHROOM3 | 0.0219 |
| *Marinilabiliaceae family* | rs11915634 | 3 | 1,452,602 | 1,517,331 | T | C | 2.99E-10 | -1.3 | 0.21 | 0.14 | CNTN6 | - | 0.0217 |
| *Marinilabiliaceae family* | rs9831278 | 3 | 98,879,786 | 98,942,990 | C | T | 2.53E-08 | -1.16 | 0.21 | 0.49 | LINC00974 | - | 0.0170 |
| *OTU10032 unclassifed Enterobacteriaceae* | rs12149695 | 16 | 27,205,994 | 27,293,886 | A | T | 1.82E-09 | 0.61 | 0.10 | 0.23 | FLJ21408 | NSMCE1, FLJ21408, KDM8 | 0.0198 |
| *OTU10032 unclassifed Enterobacteriaceae* | rs13276516 | 8 | 56,589,428 | 56,596,140 | A | G | 5.54E-09 | -0.61 | 0.10 | 0.41 | TGS1 | - | 0.0186 |
| *OTU10032 unclassifed Enterobacteriaceae* | rs17085775 | 9 | 71,165,704 | 71,167,878 | C | T | 2.06E-08 | -1.03 | 0.18 | 0.54 | C9orf71 | - | 0.0172 |
| *OTU10032 unclassifed Enterobacteriaceae* | rs249733 | 5 | 141,877,862 | 141,911,748 | T | C | 4.74E-10 | -0.65 | 0.10 | 0.68 | SPRY4 | - | 0.0212 |
| *OTU10032 unclassifed Enterobacteriaceae Species-level OTU* | rs1009634 | 12 | 4,779,313 | 4,900,344 | G | A | 7.12E-09 | -1.31 | 0.23 | 0.93 | AKAP3 | NDUFA9, GALNT8, RP11-234B24.2 | 0.0183 |
| *OTU10032 unclassifed Enterobacteriaceae Species-level OTU* | rs2318350 | 8 | 139,889,972 | 139,942,500 | T | C | 3.65E-09 | -1.15 | 0.19 | 0.95 | COL22A1 | COL22A1 | 0.0190 |
| *OTU10032 unclassifed Enterobacteriaceae Species-level OTU* | rs3925158 | 3 | 38,161,078 | 38,313,688 | C | G | 6.29E-09 | -1 | 0.17 | 0.78 | SLC22A13 | SLC22A13, MYD88, DLEC1, ACAA1, OXSR1 | 0.0185 |
| *OTU13305 Fecalibacterium Species-level OTU* | rs597205 | 1 | 112,379,026 | 112,415,622 | T | C | 7.68E-09 | -0.62 | 0.11 | 0.85 | C1orf183 | C1orf183 | 0.0183 |
| *OTU15355 Dialister Species-level OTU* | rs12442649 | 15 | 37,968,393 | 38,035,538 | G | A | 3.72E-08 | -1.49 | 0.27 | 0.85 | TMCO5A | - | 0.0166 |
| *Proteobacteria phylum* | rs9323326 | 14 | 58,476,448 | 58,532,709 | A | G | 8.76E-10 | -0.21 | 0.03 | 0.02 | SLC35F4 | C14orf37 | 0.0206 |
| *Unclassifed Acidaminococcaceae* | rs17661843 | 7 | 48,381,902 | 48,433,594 | T | C | 3.72E-14 | -1.4 | 0.18 | 0.26 | ABCA13 | ABCA13 | 0.0312 |
| *Unclassifed Acidaminococcaceae* | rs56006724 | 2 | 228,486,044 | 228,523,585 | A | G | 6.35E-10 | -0.88 | 0.14 | 0.93 | C2orf83 | C2orf83 | 0.0209 |
| *Unclassifed Acidaminococcaceae* | rs75036654 | 1 | 37,717,219 | 37,780,821 | C | T | 4.94E-10 | -1.39 | 0.22 | 0.06 | LINC01137 | - | 0.0212 |
| *Unclassifed Acidaminococcaceae* | rs986417 | 14 | 60,787,269 | 61,122,040 | C | T | 2.63E-09 | -1.4 | 0.23 | 0.47 | SIX6 | SIX6, C14orf39, SIX1 | 0.0194 |
| *Unclassifed Enterobacteriaceae* | rs938295 | 1 | 16,087,164 | 16,124,985 | C | T | 2.34E-08 | -0.49 | 0.09 | 0.76 | FBLIM1 | FBLIM1 | 0.0171 |
| *Unclassifed Erysipelotrichaceae* | rs11626933 | 14 | 90,681,816 | 90,810,659 | G | A | 1.83E-08 | -0.24 | 0.04 | 0.55 | C14orf102 | C14orf102 | 0.0173 |
| *Unclassifed Marinilabiliaceae* | rs11724031 | 4 | 77,441,448 | 77,467,405 | G | A | 2.44E-10 | -0.97 | 0.15 | 0.68 | SHROOM3 | SHROOM3 | 0.0219 |
| *Unclassifed Marinilabiliaceae* | rs11915634 | 3 | 1,452,602 | 1,517,331 | T | C | 2.99E-10 | -1.3 | 0.21 | 0.14 | CNTN7 | - | 0.0217 |
| *Unclassifed Marinilabiliaceae* | rs9831278 | 3 | 98,879,786 | 98,942,990 | C | T | 2.53E-08 | -1.16 | 0.21 | 0.49 | LINC00973 | - | 0.0170 |
| *Unclassifed Porphyromonadaceae* | rs7656342 | 4 | 9,721,358 | 9,895,176 | A | G | 2.80E-09 | 0.39 | 0.07 | 0.22 | DRD5 | SLC2A9, DRD5 | 0.0193 |
| *Unclassifed Porphyromonadaceae* | rs9291879 | 5 | 66,515,817 | 66,550,855 | C | T | 3.51E-09 | -0.58 | 0.10 | 0.08 | CD180 | - | 0.0191 |

SNP, single-nucleotide polymorphisms; Chr, chromosome; A1, effect allele; A2, non-effect allele; P, meta-analysis P value for A1; Beta, meta-analysis coeffcient for A1; SE, standard error; β-div P, P value for association with β diversity.

**Table S2. Characteristics of selected SNPs for gut metabolites.**

| **Metabolites** | **SNP** | **Chr** | **Position** | **Closest genec** | **A1** | **A2** | **MAF** | **Beta** | **SE** | **P** |
| --- | --- | --- | --- | --- | --- | --- | --- | --- | --- | --- |
| BHB | rs10496767 | 2 | 137581892 | HNMT | T | C | 0.243 | 0.20 | 0.04 | 6.67E-06 |
| BHB | rs11676084 | 2 | 169412220 | SPC25 | G | A | 0.248 | -0.21 | 0.05 | 6.77E-06 |
| BHB | rs12989808 | 2 | 112496205 | TMEM87B | A | G | 0.108 | 0.32 | 0.07 | 7.33E-06 |
| BHB | rs11898180 | 2 | 17902161 | MSGN1 | C | G | 0.377 | 0.20 | 0.05 | 9.14E-06 |
| BHB | rs17801569 | 9 | 81668013 | PSAT1 | G | A | 0.138 | -0.26 | 0.06 | 9.83E-06 |
| betaine | rs10178297 | 2 | 103998526 | MRPS9 | T | C | 0.160 | 0.21 | 0.05 | 6.74E-06 |
| betaine | rs10786317 | 10 | 83003759 | NRG3 | A | G | 0.437 | -0.16 | 0.04 | 4.72E-06 |
| betaine | rs10817686 | 9 | 116739671 | TNC | T | C | 0.471 | 0.46 | 0.10 | 1.68E-06 |
| betaine | rs10883712 | 10 | 103929295 | PPRC1 | T | G | 0.404 | 0.16 | 0.04 | 4.02E-06 |
| betaine | rs11030909 | 11 | 29933817 | FSHB | C | A | 0.089 | -0.29 | 0.06 | 7.30E-06 |
| betaine | rs11742447 | 5 | 117598798 | DMXL1 | C | T | 0.345 | 0.25 | 0.05 | 1.16E-06 |
| betaine | rs1321958 | 11 | 31005259 | DCDC1 | G | A | 0.388 | 0.15 | 0.03 | 8.12E-06 |
| betaine | rs17815398 | 18 | 47967754 | MEX3C | A | G | 0.020 | 0.84 | 0.19 | 7.19E-06 |
| betaine | rs1868264 | 2 | 239151212 | TRAF3IP1 | A | G | 0.195 | 0.19 | 0.04 | 4.92E-06 |
| betaine | rs2087307 | 12 | 10153542 | CLEC1A | T | G | 0.259 | -0.17 | 0.04 | 8.63E-06 |
| betaine | rs2879414 | 18 | 47962958 | MEX3C | A | G | 0.459 | -0.15 | 0.03 | 5.03E-06 |
| betaine | rs358538 | 19 | 37696665 | PDCD5 | A | G | 0.055 | 0.46 | 0.10 | 7.86E-06 |
| betaine | rs6862283 | 5 | 78355394 | ARSB | T | C | 0.306 | -0.19 | 0.04 | 9.57E-08 |
| carnitine | rs10821590 | 10 | 61197033 | SLC16A9 | G | A | 0.071 | 0.60 | 0.11 | 8.99E-08 |
| carnitine | rs1171610 | 10 | 61147703 | CCDC6 | T | C | 0.383 | -0.20 | 0.04 | 6.63E-08 |
| carnitine | rs1171617 | 10 | 61137188 | CCDC6 | T | G | 0.230 | -0.42 | 0.04 | 5.87E-26 |
| carnitine | rs11733138 | 4 | 22769759 | LOC643751 | G | A | 0.346 | 0.23 | 0.05 | 6.10E-06 |
| carnitine | rs16913790 | 10 | 61016188 | CCDC6 | G | A | 0.138 | 0.22 | 0.05 | 7.18E-06 |
| carnitine | rs274554 | 5 | 131752849 | LOC441108 | C | T | 0.159 | -0.21 | 0.05 | 4.68E-06 |
| carnitine | rs4656852 | 1 | 158071277 | FCRL6 | T | C | 0.053 | -0.51 | 0.11 | 8.30E-06 |
| carnitine | rs562672 | 11 | 125488674 | RPUSD4 | T | C | 0.366 | -0.23 | 0.05 | 7.39E-06 |
| carnitine | rs6108228 | 20 | 8926063 | PLCB1 | T | G | 0.406 | -0.18 | 0.04 | 2.56E-06 |
| carnitine | rs6959875 | 7 | 19788530 | TWISTNB | G | A | 0.301 | 0.27 | 0.06 | 7.58E-07 |
| carnitine | rs7606454 | 2 | 45795867 | SRBD1 | C | T | 0.139 | 0.24 | 0.05 | 9.62E-06 |
| carnitine | rs9879988 | 3 | 5284556 | ARL8B | A | G | 0.163 | 0.24 | 0.05 | 3.00E-07 |
| choline | rs10098425 | 8 | 106016051 | ZFPM2 | A | G | 0.054 | 0.46 | 0.10 | 8.93E-06 |
| choline | rs10819950 | 9 | 97975154 | SLC35D2 | A | G | 0.316 | 0.21 | 0.05 | 6.95E-06 |
| choline | rs10826197 | 10 | 59912647 | TFAM | C | A | 0.487 | 0.15 | 0.03 | 8.15E-06 |
| choline | rs12444044 | 16 | 78024057 | WWOX | A | G | 0.112 | -0.36 | 0.08 | 5.77E-06 |
| choline | rs2408564 | 12 | 90546484 | BTG1 | A | G | 0.170 | -0.25 | 0.05 | 1.41E-06 |
| choline | rs4008155 | 9 | 38478919 | ALDH1B1 | C | T | 0.156 | 0.22 | 0.05 | 4.02E-06 |
| choline | rs4312185 | 13 | 75564535 | UCHL3 | A | T | 0.427 | 0.16 | 0.04 | 7.13E-06 |
| GABA | rs10019772 | 4 | 1805344 | FGFR3 | A | G | 0.012 | -3.65 | 0.78 | 2.68E-06 |
| GABA | rs12073624 | 1 | 79235744 | IFI44 | G | A | 0.118 | -0.28 | 0.06 | 3.56E-06 |
| GABA | rs17587049 | 14 | 100761050 | DLK1 | C | A | 0.196 | 0.77 | 0.15 | 2.96E-07 |
| GABA | rs201762 | 13 | 49963837 | RNASEH2B | T | G | 0.233 | -0.19 | 0.04 | 6.88E-06 |
| GABA | rs2161525 | 19 | 7738664 | CD209 | T | C | 0.425 | 0.19 | 0.04 | 1.00E-05 |
| GABA | rs2553640 | 1 | 163733501 | RXRG | G | A | 0.090 | -0.31 | 0.07 | 9.38E-06 |
| GABA | rs2912200 | 4 | 13752463 | NKX3-2 | T | C | 0.223 | 0.20 | 0.04 | 3.39E-06 |
| GABA | rs6977081 | 7 | 150173448 | TMEM176A | G | T | 0.336 | 0.23 | 0.04 | 5.36E-09 |
| GABA | rs8051603 | 16 | 85606059 | MAP1LC3B | C | A | 0.039 | -0.58 | 0.13 | 9.13E-06 |
| GABA | rs8106338 | 19 | 2054186 | C19orf36 | T | C | 0.020 | 1.97 | 0.42 | 2.81E-06 |
| GABA | rs927428 | 10 | 123926834 | BTBD16 | G | A | 0.042 | -0.39 | 0.09 | 8.54E-06 |
| propionic acid | rs11132322 | 4 | 169713165 | FLJ31033 | C | T | 0.311 | -0.21 | 0.04 | 1.22E-06 |
| propionic acid | rs17479559 | 8 | 78981111 | C8orf70 | A | G | 0.013 | 1.18 | 0.26 | 5.74E-06 |
| propionic acid | rs6766484 | 3 | 186950433 | IGF2BP2 | C | T | 0.007 | 1.41 | 0.32 | 7.52E-06 |
| serotonin | rs10761071 | 9 | 106493148 | NIPSNAP3A | T | A | 0.186 | 0.21 | 0.04 | 1.97E-06 |
| serotonin | rs11204524 | 1 | 246089002 | OR11L1 | G | C | 0.237 | 0.30 | 0.06 | 3.43E-07 |
| serotonin | rs11715386 | 3 | 180117986 | ZMAT3 | C | T | 0.129 | -0.24 | 0.05 | 8.96E-06 |
| serotonin | rs16848753 | 2 | 213098009 | IKZF2 | A | G | 0.149 | -0.21 | 0.05 | 6.08E-06 |
| serotonin | rs17745202 | 6 | 111675717 | SLC16A10 | G | A | 0.145 | 0.22 | 0.05 | 4.39E-06 |
| serotonin | rs2014998 | 2 | 217390206 | IGFBP5 | G | A | 0.261 | -0.17 | 0.04 | 8.73E-06 |
| serotonin | rs2862078 | 3 | 154695022 | RAP2B | C | T | 0.473 | -0.16 | 0.04 | 4.31E-06 |
| serotonin | rs6908341 | 6 | 169981191 | TCTE3 | A | G | 0.120 | -0.25 | 0.06 | 8.96E-06 |
| TMAO | rs12221306 | 10 | 17041344 | RSU1 | A | T | 0.387 | -0.16 | 0.04 | 5.04E-06 |
| TMAO | rs13236413 | 7 | 156563826 | MNX1 | C | T | 0.090 | 0.41 | 0.09 | 2.23E-06 |
| TMAO | rs16917161 | 8 | 53128610 | PCMTD1 | T | A | 0.092 | 0.26 | 0.06 | 9.23E-06 |
| TMAO | rs17418675 | 2 | 55064109 | FLJ31438 | T | C | 0.341 | 0.16 | 0.04 | 9.11E-06 |
| TMAO | rs2703228 | 8 | 5863417 | ANGPT2 | C | G | 0.323 | -0.22 | 0.04 | 1.28E-07 |
| TMAO | rs3935570 | 1 | 19039958 | ALDH4A1 | G | T | 0.261 | -0.23 | 0.04 | 1.56E-07 |
| TMAO | rs4846884 | 1 | 229153698 | ARV1 | C | A | 0.156 | -0.21 | 0.05 | 6.19E-06 |
| TMAO | rs7302178 | 12 | 26899160 | C12orf11 | A | G | 0.164 | 0.24 | 0.05 | 6.40E-06 |

SNP, single-nucleotide polymorphisms; Chr, chromosome; A1, effect allele; A2, non-effect allele; SE, standard error; BHB; β-hydroxybutyric acid; GABA, γ-aminobutyric acid; TMAO, trimethylamine N-oxide.

**Table S3. Characteristics of selected SNPs for neuropsychological diseases.**

| **Disease** | **SNP** | **Chr.** | **Position** | **Closest genec** | **A1** | **A2** | **EAF** | **OR** | **P** |
| --- | --- | --- | --- | --- | --- | --- | --- | --- | --- |
| AD | rs10498633 | 14 | 92,926,952 | SLC24A4-RIN3 | G | T | 0.217 | 0.91 | 5.50E-09 |
| AD | rs10792832 | 11 | 85,867,875 | PICALM | G | A | 0.358 | 0.87 | 9.30E-26 |
| AD | rs10838725 | 11 | 47,557,871 | CELF1 | T | C | 0.316 | 1.08 | 1.10E-08 |
| AD | rs10948363 | 6 | 47,487,762 | CD2AP | A | G | 0.266 | 1.1 | 5.20E-11 |
| AD | rs11218343 | 11 | 121,435,587 | SORL1 | T | C | 0.039 | 0.77 | 9.70E-15 |
| AD | rs11771145 | 7 | 143,110,762 | EPHA1 | G | A | 0.338 | 0.9 | 1.10E-13 |
| AD | rs1476679 | 7 | 100,004,446 | ZCWPW1 | T | C | 0.287 | 0.91 | 5.60E-10 |
| AD | rs17125944 | 14 | 53,400,629 | FERMT2 | T | C | 0.092 | 1.14 | 7.90E-09 |
| AD | rs190982 | 5 | 88,223,420 | MEF2C | A | G | 0.408 | 0.93 | 3.20E-08 |
| AD | rs2718058 | 7 | 37,841,534 | NME8 | A | G | 0.373 | 0.93 | 4.80E-09 |
| AD | rs28834970 | 8 | 27,195,121 | PTK2B | T | C | 0.366 | 1.1 | 7.40E-14 |
| AD | rs35349669 | 2 | 234,068,476 | INPP5D | C | T | 0.488 | 1.08 | 3.20E-08 |
| AD | rs4147929 | 19 | 1,063,443 | ABCA7 | G | A | 0.19 | 1.15 | 1.10E-15 |
| AD | rs6656401 | 1 | 207,692,049 | CR1 | G | A | 0.197 | 1.18 | 5.70E-24 |
| AD | rs6733839 | 2 | 127,892,810 | BIN1 | C | T | 0.409 | 1.22 | 6.90E-44 |
| AD | rs7274581 | 20 | 55,018,260 | CASS4 | T | C | 0.083 | 0.88 | 2.50E-08 |
| AD | rs8093731 | 18 | 29,088,958 | DSG2 | C | T | 0.017 | 0.54 | 4.60E-08 |
| AD | rs9271192 | 6 | 32,578,530 | HLA-DRB5–HLA-DRB1 | A | C | 0.276 | 1.11 | 2.90E-12 |
| AD | rs9331896 | 8 | 27,467,686 | CLU | T | C | 0.379 | 0.86 | 2.80E-25 |
| AD | rs983392 | 11 | 59,923,508 | MS4A6A | A | G | 0.403 | 0.9 | 6.10E-16 |
| MDD | rs10149470 | 14 | 104,017,953 | BAG5 | A | G | 0.49 | 0.97 | 3.10E-09 |
| MDD | rs10950398 | 7 | 12,264,871 | TMEM106B | A | G | 0.41 | 1.03 | 2.60E-08 |
| MDD | rs10959913 | 9 | 11,544,964 | - | T | G | 0.76 | 1.03 | 5.10E-09 |
| MDD | rs11135349 | 5 | 164,523,472 | - | A | C | 0.48 | 0.97 | 1.10E-09 |
| MDD | rs115507122 | 6 | 30,737,591 | extended MHC | C | G | 0.18 | 0.96 | 3.30E-11 |
| MDD | rs11643192 | 16 | 72,214,276 | PMFBP1 | A | C | 0.41 | 1.03 | 3.40E-08 |
| MDD | rs11663393 | 18 | 50,614,732 | DCC | A | G | 0.45 | 1.03 | 1.60E-08 |
| MDD | rs116755193 | 5 | 124,251,883 | LOC101927421 | T | C | 0.38 | 0.97 | 7.00E-09 |
| MDD | rs11682175 | 2 | 57,987,593 | VRK2 | T | C | 0.52 | 0.97 | 4.70E-09 |
| MDD | rs12129573 | 1 | 73,768,366 | LINC01360 | A | C | 0.37 | 1.04 | 4.00E-12 |
| MDD | rs1226412 | 2 | 157,111,313 | LINC01876 | T | C | 0.79 | 1.03 | 2.40E-08 |
| MDD | rs12552 | 13 | 53,625,781 | OLFM4 | A | G | 0.44 | 1.04 | 6.10E-19 |
| MDD | rs12666117 | 7 | 109,105,611 | - | A | G | 0.47 | 1.03 | 1.40E-08 |
| MDD | rs12958048 | 18 | 53,101,598 | TCF4 | A | G | 0.33 | 1.03 | 3.60E-11 |
| MDD | rs1354115 | 9 | 2,983,774 | PUM3 | A | C | 0.62 | 1.03 | 2.40E-08 |
| MDD | rs1432639 | 1 | 72,813,218 | NEGR1 | A | C | 0.63 | 1.04 | 4.60E-15 |
| MDD | rs159963 | 1 | 8,504,421 | RERE | A | C | 0.56 | 0.97 | 3.20E-08 |
| MDD | rs17727765 | 17 | 27,576,962 | CRYBA1 | T | C | 0.92 | 0.95 | 8.50E-09 |
| MDD | rs1806153 | 11 | 31,850,105 | DKFZp686K1684,PAUPAR | T | G | 0.22 | 1.04 | 1.20E-09 |
| MDD | rs1833288 | 18 | 52,517,906 | RAB27B | A | G | 0.72 | 1.03 | 2.60E-08 |
| MDD | rs2389016 | 1 | 80,799,329 | - | T | C | 0.28 | 1.03 | 1.00E-08 |
| MDD | rs34215985 | 4 | 42,047,778 | SLC30A9 | C | G | 0.24 | 0.96 | 3.10E-09 |
| MDD | rs4074723 | 12 | 23,947,737 | SOX5 | A | C | 0.41 | 0.97 | 3.10E-08 |
| MDD | rs4143229 | 13 | 44,327,799 | ENOX1 | A | C | 0.92 | 0.95 | 2.50E-08 |
| MDD | rs4261101 | 1 | 90,796,053 | - | A | G | 0.37 | 0.97 | 1.00E-08 |
| MDD | rs4869056 | 5 | 166,992,078 | TENM2 | A | G | 0.63 | 0.97 | 6.80E-09 |
| MDD | rs4904738 | 14 | 42,179,732 | LRFN5 | T | C | 0.57 | 0.97 | 2.60E-09 |
| MDD | rs5758265 | 22 | 41,617,897 | L3MBTL2 | A | G | 0.28 | 1.03 | 7.60E-09 |
| MDD | rs61867293 | 10 | 106,563,924 | SORCS3 | T | C | 0.2 | 0.96 | 7.00E-10 |
| MDD | rs62099069 | 18 | 36,883,737 | MIR924HG | A | T | 0.42 | 0.97 | 1.30E-08 |
| MDD | rs7029033 | 9 | 126,682,068 | DENND1A | T | C | 0.07 | 1.05 | 2.70E-08 |
| MDD | rs7198928 | 16 | 7,666,402 | RBFOX1 | T | C | 0.62 | 1.03 | 1.00E-08 |
| MDD | rs7200826 | 16 | 13,066,833 | SHISA9 | T | C | 0.25 | 1.03 | 2.40E-08 |
| MDD | rs7430565 | 3 | 158,107,180 | RSRC1 | A | G | 0.58 | 0.97 | 2.90E-09 |
| MDD | rs7856424 | 9 | 119,733,595 | ASTN2 | T | C | 0.29 | 0.97 | 8.50E-09 |
| MDD | rs8025231 | 15 | 37,648,402 | - | A | C | 0.57 | 0.97 | 2.40E-12 |
| MDD | rs8063603 | 16 | 6,310,645 | RBFOX1 | A | G | 0.65 | 0.97 | 6.90E-09 |
| MDD | rs915057 | 14 | 64,686,207 | SYNE2 | A | G | 0.42 | 0.97 | 7.60E-10 |
| MDD | rs9402472 | 6 | 99,566,521 | C6orf168 | A | G | 0.24 | 1.03 | 2.80E-08 |
| MDD | rs9427672 | 1 | 197,754,741 | DENND1B | A | G | 0.24 | 0.97 | 3.10E-08 |
| SCZ | rs1006737 | 12 | 2,345,295 | CACNA1C | A | G | 0.332 | 1.103 | 5.22E-12 |
| SCZ | rs10789369 | 1 | 73,824,909 | LINC01360 | A | G | 0.383 | 1.095 | 3.64E-10 |
| SCZ | rs114002140 | 6 | 32,431,962 | MPHOSPH9, C12orf65 | A | G | 0.763 | 1.167 | 9.14E-14 |
| SCZ | rs11532322 | 12 | 123,731,423 | - | A | G | 0.318 | 1.094 | 2.28E-08 |
| SCZ | rs1198588 | 1 | 98,552,832 | RP11-586K2.1 | A | T | 0.214 | 0.889 | 1.72E-12 |
| SCZ | rs11995572 | 8 | 89,592,083 | ZEB2 | T | G | 0.135 | 1.12 | 3.33E-08 |
| SCZ | rs12991836 | 2 | 145,141,541 | SDCCAG8, AKT3 | A | C | 0.652 | 0.922 | 1.19E-08 |
| SCZ | rs14403 | 1 | 243,663,893 | SDCCAG8 | T | C | 0.227 | 0.91 | 1.80E-08 |
| SCZ | rs1538774 | 1 | 243,544,827 | CTC-436P18.1, CTC-436P18.3 | C | G | 0.26 | 0.917 | 2.53E-08 |
| SCZ | rs171748 | 5 | 60,499,131 | - | A | G | 0.471 | 1.078 | 3.78E-08 |
| SCZ | rs17504622 | 5 | 152,654,479 | CACNB2 | T | C | 0.05 | 1.238 | 2.65E-09 |
| SCZ | rs17691888 | 10 | 18,734,528 | QPCT | A | G | 0.114 | 0.862 | 1.27E-10 |
| SCZ | rs2373000 | 2 | 37,592,628 | MAU2 | T | C | 0.402 | 1.087 | 6.78E-09 |
| SCZ | rs2905424 | 19 | 19,473,445 | LINC01470 | T | C | 0.348 | 1.092 | 3.44E-09 |
| SCZ | rs2910032 | 5 | 152,540,354 | FTCDNL1 | T | C | 0.531 | 0.925 | 4.12E-08 |
| SCZ | rs2949006 | 2 | 200,715,388 | TSNARE1 | T | G | 0.192 | 1.102 | 1.21E-08 |
| SCZ | rs4129585 | 8 | 143,312,933 | ITIH3 | A | C | 0.439 | 1.091 | 2.19E-10 |
| SCZ | rs4687552 | 3 | 52,838,402 | - | T | C | 0.641 | 1.086 | 1.16E-08 |
| SCZ | rs4801131 | 18 | 52,752,700 | MAD1L1 | T | C | 0.418 | 0.925 | 1.22E-08 |
| SCZ | rs6461049 | 7 | 2,017,445 | SLCO6A1 | T | C | 0.571 | 1.107 | 5.93E-13 |
| SCZ | rs6878284 | 5 | 101,769,726 | C10orf32-ASMT, C10orf32, AS3MT | T | C | 0.637 | 0.92 | 9.03E-09 |
| SCZ | rs7085104 | 10 | 104,628,873 | C2orf82, NGEF | A | G | 0.645 | 1.11 | 3.68E-13 |
| SCZ | rs778371 | 2 | 233,743,109 | - | A | G | 0.719 | 0.92 | 1.51E-08 |
| SCZ | rs7940866 | 11 | 130,817,579 | HLA-DRB9 | A | T | 0.513 | 0.921 | 1.83E-09 |

SNP, single-nucleotide polymorphisms; OR, odds ratio; CI, confidence internal; AD, Alzheimer’s disease; MDD, major depressive; SCZ, schizophrenia.

**Table S4. Description of the diagnostic assessment for neuropsychological diseases.**

| **(a)Alzheimer's disease (AD)** |  |  |
| --- | --- | --- |
| **Study/Consortium** | **AD cases** | **Diagnostic criteria for AD** |
| **ADGC** | | |
| ACT | 566 | NINCDS-ADRDA criteria for possible or probable AD |
| ADC | 2512 | DSM-IV criteria or CDR ≥1 (70% autopsy-confirmed) |
| ADNI | 268 | NINCDS-ADRDA criteria for probable AD |
| GenADA | 669 | NINCDS-ADRDA and DSM-IV criteria for probable AD |
| Mayo-Clinic | 728 | NINCDS-ADRDA criteria for possible or probable AD (34% autopsy-confirmed) |
| MIRAGE | 509 | NINCDS-ADRDA criteria for possible or probable AD |
| NCRAD/NIA-LOAD | 1811 | NINCDS-ADRDA criteria for possible, probable or definite AD |
| OHSU | 131 | Autopsy-confirmed AD |
| ROS/MAP | 291 | Clinical criteria for AD |
| TGEN2 | 129 | Possible or probable AD (autopsy-confirmed) |
| UM/VU/MSSM | 1070 | NINCDS-ADRDA criteria for possible or probable AD (34% autopsy-confirmed) |
| UP | 1271 | NINCDS-ADRDA criteria for possible or probable AD |
| WU | 318 | Standard criteria |
| **CHARGE** | | |
| AGES-RS | 78 | NINCDS-ADRDA criteria for possible or probable AD |
| CHS | 421 | NINCDS-ADRDA criteria for possible or probable AD |
| FHS | 183 | NINCDS-ADRDA criteria for possible, probable or definite AD |
| Rotterdam study | 633 | NINCDS-ADRDA criteria for possible, probable or definite AD |
| **EADI** | 2243 | NINCDS-ADRDA criteria for possible or probable AD |
| **GERAD** | 3177 | NINCDS-ADRDA and DSM-IV criteria for probable AD and CERAD criteria for definite AD |
| **(b) Major depression disorder (MDD)** | |  |
| **Study/Consortium** | **MDD cases** | **Diagnostic criteria for MDD (lifetime)** |
| PGC29 | 16823 | DSM-IV MDD |
| deCODE | 1980 | DSM-III, ICD-9 or ICD-10 MDD (recurrent or moderate-severe single episode) |
| GenScotland | 997 | DSM-IV MDD |
| GERA | 7162 | ICD-9 MDD on ≥2 separate medical visits |
| iPSYCH | 18629 | ICD-10 MDD |
| UK Biobank | 14260 | ICD-10 MDD inpatient, saw psychiatrist for anxiety or MDD, depression or anhedonia ≥2 weeks |
| 23andMe | 75607 | MDD (diagnosed with clinical depression or depression diagnosed by a doctor) |
| **(c) Schizophrenia (SCZ)** |  |  |
| **Study/Consortium** | **SCZ cases** | **Diagnostic criteria for SCZ** |
| Sweden | 5001 | SCZ (≥2 hospitalizations with a discharge diagnosis of schizophrenia) |
| PGC | 8832 | SCZ (≥2 hospitalizations with a discharge diagnosis of schizophrenia) |
| Replication | 7413 | SCZ (≥2 hospitalizations with a discharge diagnosis of schizophrenia) |

ADGC: Alzheimer Disease Genetics Consortium; ACT: ACT/eMERGE Studies; NINCDS-ADRDA: National Institute of Neurological and Communicative Disorders and Stroke and the Alzheimer’s Disease and Related Disorders Association; ADC: NIA ADC Samples; DSM-IV: Diagnostic and Statistical Manual of Mental Disorders,4th Edition; CDR: Clinical Dementia Rating; OHSU: Oregon Health and Science University; ADNI: ADNI Study; NCRAD/NIA-LOAD: NCRAD/NIA-LOAD Family Study; UM/VU/MSSM: University of Miami/Vanderbilt University/Mt. Sinai School of Medicine; UP: University of Pittsburgh; WU: Washington University; CHARGE: Cohort for Heart and Ageing Research in Genomic Epidemiology; CHS: Cardiovascular Health Study; FHS: Framingham Heart Study; EADI: European Alzheimer’s Disease Initiative; GERAD: Genetic and Environmental Risk in Alzheimer’s Disease; CERAD: Consortium to Establish a Registry for Alzheimer's Disease; GERA, Genetic Epidemiology Research on Adult Health and Aging; PGC, Psychiatric Genomics Consortium; AD, Alzheimer’s disease; MDD, major depressive; SCZ, schizophrenia.

**Table S5. Associations between genetically predicted gut microbiota and neuropsychological diseases in sensitivity analyses.**

| **Gut microbiota** | **Diseases** | | **Methods** | **No. of SNPs** | **OR(95%CI)** | **P-value** | **MR-Egger Intercept** | **P-value fot the Intercept** |
| --- | --- | --- | --- | --- | --- | --- | --- | --- |
| *Bacilli class* | AD | | Weighted mode | 6 | 1.01(0.90,1.14) | 0.863 | -0.002 | 0.960 |
|  |  | | Weighted median | 6 | 1.00(0.93,1.07) | 0.899 |  |  |
|  |  | | MR Egger | 6 | 0.99(0.76,1.30) | 0.964 |  |  |
|  | SCZ | | Weighted mode | 6 | 1.00(0.89,1.11) | 0.937 | -0.016 | 0.670 |
|  |  | | Weighted median | 6 | 0.98(0.90,1.06) | 0.622 |  |  |
|  |  | | MR Egger | 6 | 1.02(0.79,1.32) | 0.890 |  |  |
| *Lactobacillales order* | | AD | Weighted mode | 6 | 0.96(0.87,1.06) | 0.455 | 0.003 | 0.934 |
|  |  |  | Weighted median | 6 | 0.97(0.90,1.03) | 0.311 |  |  |
|  |  |  | MR Egger | 6 | 0.97(0.77,1.22) | 0.823 |  |  |
|  |  | MDD | Weighted mode | 5 | 1.00(0.94,1.06) | 0.964 | 0.012 | 0.578 |
|  |  |  | Weighted median | 5 | 1.01(0.97,1.05) | 0.643 |  |  |
|  |  |  | MR Egger | 5 | 0.97(0.84,1.11) | 0.659 |  |  |
|  |  | SCZ | Weighted mode | 6 | 1.03(0.92,1.17) | 0.624 | -0.050 | 0.182 |
|  |  |  | Weighted median | 6 | 1.02(0.95,1.10) | 0.598 |  |  |
|  |  |  | MR Egger | 6 | 1.17(0.93,1.47) | 0.250 |  |  |
| *Marinilabiliaceae family* | | AD | Weighted mode | 3 | 1.01(0.95,1.07) | 0.838 | 0.073 | 0.510 |
|  |  |  | Weighted median | 3 | 1.00(0.95,1.04) | 0.904 |  |  |
|  |  |  | MR Egger | 3 | 0.91(0.77,1.07) | 0.454 |  |  |
|  |  | MDD | Weighted mode | 4 | 1.00(0.97,1.03) | 0.910 | -0.044 | 0.399 |
|  |  |  | Weighted median | 4 | 1.01(0.99,1.03) | 0.255 |  |  |
|  |  |  | MR Egger | 4 | 1.06(0.97,1.15) | 0.329 |  |  |
|  |  | SCZ | Weighted mode | 4 | 1.03(0.98,1.07) | 0.350 | 0.008 | 0.900 |
|  |  |  | Weighted median | 4 | 1.02(0.99,1.06) | 0.135 |  |  |
|  |  |  | MR Egger | 4 | 1.02(0.90,1.14) | 0.821 |  |  |
| *OTU10032 unclassifed Enterobacteriaceae* | | AD | Weighted mode | 5 | 0.97(0.93,1.02) | 0.281 | -0.035 | 0.462 |
|  |  |  | Weighted median | 5 | 0.98(0.95,1.01) | 0.259 |  |  |
|  |  |  | MR Egger | 5 | 1.04(0.92,1.18) | 0.582 |  |  |
|  |  | MDD | Weighted mode | 3 | 1.00(0.97,1.03) | 0.966 | 0.007 | 0.910 |
|  |  |  | Weighted median | 3 | 1.00(0.98,1.02) | 0.799 |  |  |
|  |  |  | MR Egger | 3 | 0.99(0.86,1.13) | 0.873 |  |  |
|  |  | SCZ | Weighted mode | 5 | 1.01(0.97,1.05) | 0.715 | 0.064 | 0.239 |
|  |  |  | Weighted median | 5 | 1.01(0.98,1.05) | 0.510 |  |  |
|  |  |  | MR Egger | 5 | 0.93(0.82,1.06) | 0.367 |  |  |
| *OTU10032 unclassifed Enterobacteriaceae Species-level OTU* | | AD | Weighted mode | 3 | 0.98(0.94,1.02) | 0.386 | 0.088 | 0.701 |
|  |  |  | Weighted median | 3 | 0.98(0.94,1.01) | 0.203 |  |  |
|  |  |  | MR Egger | 3 | 0.90(0.66,1.22) | 0.614 |  |  |
|  |  | SCZ | Weighted mode | 3 | 0.97(0.92,1.03) | 0.457 | 0.332 | 0.313 |
|  |  |  | Weighted median | 3 | 0.98(0.94,1.03) | 0.491 |  |  |
|  |  |  | MR Egger | 3 | 0.74(0.54,1.01) | 0.313 |  |  |
| *Unclassifed Acidaminococcaceae* | | AD | Weighted mode | 4 | 1.01(0.97,1.04) | 0.674 | 0.027 | 0.695 |
|  |  |  | Weighted median | 4 | 1.01(0.98,1.03) | 0.551 |  |  |
|  |  |  | MR Egger | 4 | 0.98(0.89,1.08) | 0.706 |  |  |
|  |  | MDD | Weighted mode | 4 | 1.00(0.98,1.01) | 0.635 | 0.057 | 0.438 |
|  |  |  | Weighted median | 4 | 1.00(0.98,1.01) | 0.472 |  |  |
|  |  |  | MR Egger | 4 | 0.95(0.86,1.04) | 0.377 |  |  |
|  |  | SCZ | Weighted mode | 4 | 1.02(0.99,1.06) | 0.299 | 0.025 | 0.735 |
|  |  |  | Weighted median | 4 | 1.01(0.99,1.04) | 0.280 |  |  |
|  |  |  | MR Egger | 4 | 0.99(0.89,1.10) | 0.908 |  |  |
| *Unclassifed Marinilabiliaceae* | | AD | Weighted mode | 3 | 1.01(0.94,1.07) | 0.845 | 0.073 | 0.510 |
|  |  |  | Weighted median | 3 | 1.00(0.95,1.04) | 0.904 |  |  |
|  |  |  | MR Egger | 3 | 0.91(0.77,1.07) | 0.454 |  |  |
|  |  | MDD | Weighted mode | 4 | 1.00(0.96,1.03) | 0.917 | -0.044 | 0.399 |
|  |  |  | Weighted median | 4 | 1.01(0.99,1.03) | 0.234 |  |  |
|  |  |  | MR Egger | 4 | 1.06(0.97,1.15) | 0.329 |  |  |
|  |  | SCZ | Weighted mode | 4 | 1.03(0.98,1.07) | 0.322 | 0.008 | 0.900 |
|  |  |  | Weighted median | 4 | 1.02(0.99,1.06) | 0.153 |  |  |
|  |  |  | MR Egger | 4 | 1.02(0.90,1.14) | 0.821 |  |  |

SNP, single-nucleotide polymorphisms; OR, odds ratio; CI, confidence internal; AD, Alzheimer’s disease; MDD, major depressive; SCZ, schizophrenia. OR (95% CI) means risk of neuropsychological diseases per relative abundance of gut microbiota.

**Table S6. Associations between genetically predicted gut microbiota and neuropsychological diseases in a leave-one-out approach.**

| **Gut microbiota** | **Excluded SNP** | **AD results** | | **MDD results** | | **SCZ results** | |
| --- | --- | --- | --- | --- | --- | --- | --- |
|  |  | **OR(95%CI)** | **P-value** | **OR(95%CI)** | **P-value** | **OR(95%CI)** | **P-value** |
| *Bacilli class* | rs10928827 | 1.00(0.93,1.06) | 0.925 | - | - | 0.98(0.92,1.05) | 0.559 |
| *Bacilli class* | rs148330122 | 0.98(0.92,1.05) | 0.571 | - | - | 0.96(0.90,1.03) | 0.260 |
| *Bacilli class* | rs2071199 | 1.00(0.94,1.07) | 0.977 | - | - | 0.96(0.89,1.02) | 0.190 |
| *Bacilli class* | rs479105 | 0.99(0.92,1.06) | 0.717 | - | - | 0.97(0.91,1.04) | 0.378 |
| *Bacilli class* | rs7083345 | 0.97(0.90,1.04) | 0.348 | - | - | 0.95(0.89,1.02) | 0.133 |
| *Bacilli class* | rs7646786 | 0.98(0.92,1.05) | 0.591 | - | - | 0.95(0.89,1.02) | 0.160 |
| *Lactobacillales order* | rs10928827 | 0.99(0.93,1.05) | 0.752 | - | - | 0.99(0.93,1.06) | 0.862 |
| *Lactobacillales order* | rs1362404 | 0.99(0.93,1.05) | 0.688 | 1.02(0.98,1.05) | 0.328 | 0.98(0.91,1.06) | 0.669 |
| *Lactobacillales order* | rs59042687 | 0.99(0.93,1.05) | 0.620 | 1.01(0.96,1.05) | 0.772 | 0.99(0.92,1.06) | 0.751 |
| *Lactobacillales order* | rs62295801 | 0.98(0.92,1.04) | 0.424 | 1.00(0.96,1.05) | 0.833 | 0.96(0.89,1.03) | 0.252 |
| *Lactobacillales order* | rs7083345 | 0.97(0.91,1.03) | 0.288 | 0.99(0.96,1.03) | 0.757 | 0.97(0.90,1.04) | 0.399 |
| *Lactobacillales order* | rs7113056 | 0.99(0.93,1.05) | 0.669 | 1.02(0.97,1.06) | 0.439 | 0.96(0.89,1.03) | 0.232 |
| *Marinilabiliaceae family* | rs11724031 | - | - | 1.02(1.00,1.04) | 0.041 | 1.02(0.99,1.06) | 0.208 |
| *Marinilabiliaceae family* | rs11915634 | 1.01(0.96,1.05) | 0.738 | 1.00(0.98,1.03) | 0.838 | 1.03(0.99,1.06) | 0.157 |
| *Marinilabiliaceae family* | rs9831278 | 0.97(0.91,1.04) | 0.428 | 1.01(0.98,1.03) | 0.591 | 1.03(0.99,1.06) | 0.124 |
| *Marinilabiliaceae family* | rs9996716 | 0.97(0.91,1.03) | 0.300 | 1.01(0.98,1.05) | 0.355 | 1.02(0.99,1.06) | 0.177 |
| *OTU10032 unclassifed Enterobacteriaceae* | rs12149695 | 0.99(0.96,1.01) | 0.328 | 0.99(0.97,1.01) | 0.169 | 1.03(0.99,1.07) | 0.136 |
| *OTU10032 unclassifed Enterobacteriaceae* | rs13276516 | 0.99(0.96,1.02) | 0.569 | - | - | 1.02(0.98,1.06) | 0.291 |
| *OTU10032 unclassifed Enterobacteriaceae* | rs17085775 | 0.98(0.95,1.01) | 0.121 | 1.00(0.96,1.03) | 0.808 | 1.03(0.99,1.07) | 0.111 |
| *OTU10032 unclassifed Enterobacteriaceae* | rs249733 | 0.99(0.97,1.02) | 0.645 | 1.00(0.99,1.02) | 0.661 | 1.03(1.00,1.07) | 0.074 |
| *OTU10032 unclassifed Enterobacteriaceae* | rs938295 | 0.98(0.96,1.01) | 0.240 | - | - | 1.02(0.99,1.05) | 0.234 |
| *OTU10032 unclassifed Enterobacteriaceae Species-level OTU* | rs1009634 | 0.98(0.94,1.01) | 0.209 | - | - | 1.01(0.95,1.08) | 0.777 |
| *OTU10032 unclassifed Enterobacteriaceae Species-level OTU* | rs2318350 | 0.97(0.93,1.01) | 0.122 | - | - | 1.01(0.94,1.09) | 0.765 |
| *OTU10032 unclassifed Enterobacteriaceae Species-level OTU* | rs3925158 | 0.97(0.93,1.01) | 0.110 | - | - | 0.97(0.93,1.01) | 0.195 |
| *Unclassifed Acidaminococcaceae* | rs17661843 | 1.01(0.98,1.03) | 0.528 | 0.99(0.96,1.02) | 0.464 | 1.01(0.98,1.03) | 0.658 |
| *Unclassifed Acidaminococcaceae* | rs56006724 | 1.00(0.97,1.02) | 0.858 | 0.99(0.96,1.01) | 0.212 | 1.01(0.99,1.04) | 0.394 |
| *Unclassifed Acidaminococcaceae* | rs75036654 | 1.00(0.98,1.02) | 0.926 | 1(0.99,1.01) | 0.889 | 1.02(0.99,1.04) | 0.151 |
| *Unclassifed Acidaminococcaceae* | rs986417 | 1.00(0.97,1.02) | 0.857 | 0.99(0.96,1.02) | 0.457 | 1.02(0.99,1.04) | 0.184 |
| *Unclassifed Marinilabiliaceae* | rs11724031 | - | - | 1.02(1.00,1.04) | 0.041 | 1.02(0.99,1.06) | 0.208 |
| *Unclassifed Marinilabiliaceae* | rs11915634 | 1.01(0.96,1.05) | 0.738 | 1.00(0.98,1.03) | 0.838 | 1.03(0.99,1.06) | 0.157 |
| *Unclassifed Marinilabiliaceae* | rs9831278 | 0.97(0.91,1.04) | 0.428 | 1.01(0.98,1.03) | 0.591 | 1.03(0.99,1.06) | 0.124 |
| *Unclassifed Marinilabiliaceae* | rs9996716 | 0.97(0.91,1.03) | 0.300 | 1.01(0.98,1.05) | 0.355 | 1.02(0.99,1.06) | 0.177 |

SNP, single-nucleotide polymorphisms; OR, odds ratio; CI, confidence internal; AD, Alzheimer’s disease; MDD, major depressive; SCZ, schizophrenia. OR (95% CI) means risk of neuropsychological diseases per 1-allele increase in single nucleotide polymorphisms related to greater relative abundance of gut microbiota.

**Table S7. Associations between genetically predicted metabolites and neuropsychological diseases using IVW method.**

| **Metabolites** | **Diseases** | **No. of SNPs** | **OR(95%CI)** | **P-value** |
| --- | --- | --- | --- | --- |
| BHB | AD | 5 | 0.97(0.90,1.04) | 0.371 |
|  | MDD | 3 | 0.98(0.93,1.03) | 0.397 |
|  | SCZ | 5 | 1.07(0.88,1.30) | 0.510 |
| GABA | AD | 9 | 0.96(0.92,1.00) | 0.034 |
|  | MDD | 8 | 1.01(0.99,1.03) | 0.484 |
|  | SCZ | 11 | 0.98(0.93,1.03) | 0.388 |
| Propionic acid | AD | 3 | 0.97(0.91,1.04) | 0.407 |
|  | MDD | 2 | 1.01(0.94,1.09) | 0.708 |
|  | SCZ | 3 | 0.95(0.88,1.03) | 0.239 |
| serotonin | AD | 8 | 0.99(0.93,1.06) | 0.760 |
|  | MDD | 7 | 0.97(0.93,1.00) | 0.057 |
|  | SCZ | 8 | 1.07(1.00,1.15) | 0.047 |
| TMAO | AD | 8 | 0.99(0.93,1.05) | 0.697 |
|  | MDD | 8 | 0.98(0.96,1.01) | 0.255 |
|  | SCZ | 9 | 0.99(0.96,1.02) | 0.343 |
| Betaine | AD | 13 | 1.02(0.97,1.07) | 0.353 |
|  | MDD | 16 | 1.01(0.98,1.04) | 0.460 |
|  | SCZ | 14 | 1.04(0.98,1.11) | 0.201 |
| Carnitine | AD | 12 | 1.02(0.98,1.07) | 0.310 |
|  | MDD | 12 | 1.01(0.99,1.03) | 0.620 |
|  | SCZ | 13 | 1.00(0.96,1.05) | 0.993 |
| Choline | AD | 7 | 1.02(0.95,1.09) | 0.650 |
|  | MDD | 8 | 1.01(0.98,1.04) | 0.511 |
|  | SCZ | 8 | 0.99(0.92,1.06) | 0.698 |

SNP, single-nucleotide polymorphisms; OR, odds ratio; CI, confidence internal; AD, Alzheimer’s disease; MDD, major depressive; SCZ, schizophrenia; BHB, β-hydroxybutyrate; GABA, γ-aminobutyric acid; TMAO, trimethylamine-N-oxide. OR (95% CI) means risk of neuropsychological diseases per 10 units increase in genetically predicted gut metabolites

**Table S8. MR Power calculation for detecting significant (P < 7.6×10^-4^) causal effect (OR = 1.2) of gut microbiome on the risk of AD, MDD, and SCZ.**

| **Core gut microbiota** | **Variance explained** | **Power calculation** | | |
| --- | --- | --- | --- | --- |
|  |  | **AD** | **SCZ** | **MDD** |
| *Actinobacteria class* | 0.0209 | 0.56 | 0.42 | 1.00 |
| *Actinobacteria phylum* | 0.0209 | 0.56 | 0.42 | 1.00 |
| *Bacilli class* | 0.1117 | 1.00 | 1.00 | 1.00 |
| *Blautia genus* | 0.0409 | 0.94 | 0.85 | 1.00 |
| *Enterobacteriaceae family* | 0.0239 | 0.65 | 0.51 | 1.00 |
| *Enterobacteriales order* | 0.0239 | 0.65 | 0.51 | 1.00 |
| *Erysipelotrichaceae family* | 0.0408 | 0.94 | 0.85 | 1.00 |
| *Erysipelotrichales order* | 0.0408 | 0.94 | 0.85 | 1.00 |
| *Erysipelotrichia class* | 0.0408 | 0.94 | 0.85 | 1.00 |
| *EscherichiaShigella* | 0.0170 | 0.42 | 0.30 | 1.00 |
| *Gammaproteobacteria class* | 0.0377 | 0.91 | 0.81 | 1.00 |
| *Lactobacillales order* | 0.1255 | 1.00 | 1.00 | 1.00 |
| *Marinilabiliaceae family* | 0.0606 | 1.00 | 0.98 | 1.00 |
| *OTU10032 unclassifed Enterobacteriaceae* | 0.1326 | 1.00 | 1.00 | 1.00 |
| *OTU13305 Fecalibacterium Species-level OTU* | 0.0183 | 0.47 | 0.34 | 1.00 |
| *OTU15355 Dialister Species-level OTU* | 0.0166 | 0.41 | 0.29 | 1.00 |
| *Proteobacteria phylum* | 0.0206 | 0.55 | 0.41 | 1.00 |
| *Unclassifed Acidaminococcaceae* | 0.0927 | 1.00 | 1.00 | 1.00 |
| *Unclassifed Enterobacteriaceae* | 0.0171 | 0.42 | 0.31 | 1.00 |
| *Unclassifed Erysipelotrichaceae* | 0.0173 | 0.43 | 0.31 | 1.00 |
| *Unclassifed Marinilabiliaceae* | 0.0606 | 1.00 | 0.98 | 1.00 |
| *Unclassifed Porphyromonadaceae* | 0.0384 | 0.92 | 0.82 | 1.00 |

AD, Alzheimer’s disease; MDD, major depressive; SCZ, schizophrenia.

**Table S9. Effect estimates for association of genetically predicted neuropsychological diseases with gut microbiota using four Mendelian randomization methods.**

| **Diseases** | **Gut microbiota** | **Methods** | **No. of SNPs** | **Beta** | **SE** | **P-value** | **MR-Egger Intercept** | **P-value for the Intercept** |
| --- | --- | --- | --- | --- | --- | --- | --- | --- |
| AD | *Erysipelotrichaceae_FAMILY* | Weighted mode | 11 | -0.151 | 0.236 | 0.301 | -0.039 | 0.289 |
|  |  | Weighted median | 11 | -0.223 | 0.110 | 0.076 |  |  |
|  |  | MR Egger | 11 | 0.191 | 0.193 | 0.525 |  |  |
|  |  | IVW | 11 | -0.274 | 0.090 | 0.003 |  |  |
|  | *Erysipelotrichales_ORDER* | Weighted mode | 11 | -0.151 | 0.236 | 0.301 | -0.039 | 0.289 |
|  |  | Weighted median | 11 | -0.223 | 0.110 | 0.076 |  |  |
|  |  | MR Egger | 11 | 0.191 | 0.193 | 0.525 |  |  |
|  |  | IVW | 11 | -0.274 | 0.090 | 0.003 |  |  |
|  | *Erysipelotrichia_CLASS* | Weighted mode | 11 | -0.151 | 0.236 | 0.301 | -0.039 | 0.289 |
|  |  | Weighted median | 11 | -0.223 | 0.110 | 0.076 |  |  |
|  |  | MR Egger | 11 | 0.191 | 0.193 | 0.525 |  |  |
|  |  | IVW | 11 | -0.274 | 0.090 | 0.003 |  |  |
|  | *unclassified_Porphyromonadaceae* | Weighted mode | 11 | 0.663 | 0.304 | 0.054 | -0.011 | 0.852 |
|  |  | Weighted median | 11 | 0.507 | 0.242 | 0.036 |  |  |
|  |  | MR Egger | 11 | 0.438 | 0.530 | 0.431 |  |  |
|  |  | IVW | 11 | 0.351 | 0.170 | 0.040 |  |  |
| MDD | *unclassified_Clostridiales* | Weighted mode | 35 | 0.688 | 0.611 | 0.269 | -0.011 | 0.834 |
|  |  | Weighted median | 35 | 0.652 | 0.306 | 0.033 |  |  |
|  |  | MR Egger | 35 | 1.054 | 1.767 | 0.555 |  |  |
|  |  | IVW | 35 | 0.577 | 0.241 | 0.017 |  |  |
|  | *OTU16802_Bacteroides* | Weighted mode | 35 | 0.049 | 0.988 | 0.961 | -0.197 | 0.022 |
|  |  | Weighted median | 35 | 0.399 | 0.471 | 0.399 |  |  |
|  |  | MR Egger | 35 | 6.061 | 2.665 | 0.030 |  |  |
|  |  | IVW | 35 | 0.842 | 0.386 | 0.029 |  |  |
|  | *unclassified_Prevotellaceae* | Weighted mode | 35 | 1.095 | 1.337 | 0.420 | 0.062 | 0.537 |
|  |  | Weighted median | 35 | 1.105 | 0.654 | 0.092 |  |  |
|  |  | MR Egger | 35 | 1.793 | 3.301 | 0.595 |  |  |
|  |  | IVW | 35 | 0.978 | 0.464 | 0.035 |  |  |
| SCZ | *OTU10589_unclassified_Enterobacteriaceae* | Weighted mode | 14 | -0.041 | 0.488 | 0.941 | 0.050 | 0.562 |
|  |  | Weighted median | 14 | 0.068 | 0.312 | 0.823 |  |  |
|  |  | MR Egger | 14 | -0.799 | 0.954 | 0.421 |  |  |
|  |  | IVW | 14 | 0.457 | 0.220 | 0.037 |  |  |
|  | *unclassified_Erysipelotrichaceae* | Weighted mode | 14 | -0.446 | -0.035 | 0.156 | 0.052 | 0.293 |
|  |  | Weighted median | 14 | -0.261 | -0.011 | 0.128 |  |  |
|  |  | MR Egger | 14 | -0.734 | -0.087 | 0.206 |  |  |
|  |  | IVW | 14 | -0.248 | -0.019 | 0.045 |  |  |

SNP, single-nucleotide polymorphisms; OR, odds ratio; CI, confidence internal; IVW, inverse variance weighting; AD, Alzheimer’s disease; MDD, major depressive; SCZ, schizophrenia. Beta(SE) means relative abundance of gut microbiota per each 1 unit higher log odds of neuropsychological diseases.
